# Exploring Molecular and Genetic Differences in *Angelica biserrata* Roots Under Environmental Changes

**DOI:** 10.3390/ijms26083894

**Published:** 2025-04-20

**Authors:** Chaogui Hu, Qian Li, Xiaoqin Ding, Kan Jiang, Wei Liang

**Affiliations:** State Key Laboratory of Aridland Crop Science, College of Agronomy, Gansu Agricultural University, Lanzhou 730070, China; hcg1949hcg@outlook.com (C.H.); dingxq1026@126.com (X.D.); jiangk@gsau.edu.cn (K.J.); liangw@gsau.edu.cn (W.L.)

**Keywords:** *Angelica biserrata* roots, molecular, genetic information, multi-omics, quality variation

## Abstract

*Angelica biserrata* (Shan et Yuan) Yuan et Shan (*A. biserrata*) roots, a widely distributed medicinal crop with intraspecific diversity, exhibits significant variability in coumarin content across habitats. This study integrated metabolomics and transcriptomics to dissect the spatial heterogeneity in metabolite profiles and gene expression, revealing the mechanisms driving coumarin biosynthesis divergence. By synthesizing climate-related big data with machine learning and Bayesian-optimized deep learning models, we identified key environmental drivers and predicted optimal cultivation conditions. The key findings were as follows: (1) differential regions most strongly influenced coumarin; (2) upstream genes (such as *PAL-1*, *PAL-2*, *BGLU44,* etc.) modulated downstream coumarin metabolites; (3) elevation (Elev) and warmest quarter temperature (Bio10) dominated coumarin variation, whereas May solar radiation (Srad5) and precipitation seasonality (Bio15) controlled transcriptomic reprogramming; (4) the optimized environment for bioactive compounds included mean annual temperature (Bio1) = 9.99 °C, annual precipitation (Bio12) = 1493 mm, Elev = 1728 m, cumulative solar radiation = 152,643 kJ·m^−2^·day^−1^, and soil organic carbon = 11,883 g·kg^−1^. This study aimed to clarify the biological characteristics and differential regulatory mechanisms of *A. biserrata* roots in different habitats, establish a theoretical framework for understanding the molecular mechanisms controlling metabolic changes under various habitats, and contribute to elucidating the formation of active constituents while facilitating their effective utilization.

## 1. Introduction

Medicinal plants, as critical resources for healthcare and industrial applications, exhibit close correlations between their growth environments and medicinal quality, bioactive compound accumulation, and ecological adaptability [1]. In recent years, the impacts of environmental factors on the distribution, physiological metabolism, and pharmacologically active components of medicinal plants have emerged as a research focus in interdisciplinary fields spanning ecology, pharmacology, and agronomy, driven by global climate change, intensified environmental pollution, and the growing demand for plant-derived medicinal resources. Studies demonstrate that distinct growth environments significantly influence the growth cycles and secondary metabolite synthesis in medicinal plants. For example, Zhou et al. revealed that altitudinal gradients induced marked variations in the flavonoid metabolites of *Agriophyllum squarrosum* [2]. Similarly, Hosseini et al. reported that drought conditions significantly affected glycyrrhizin synthesis licorice (*Glycyrrhiza glabra* L.) [3]. However, secondary metabolites in medicinal plants serve not only as crucial products for environmental adaptation but also as key bioactive compounds. Consequently, investigating the relationship between secondary metabolites and ecological factors is essential for ecological cultivation and the quality control of medicinal plants from the source.

The root of *Angelica biserrata* (Shan et Yuan) Yuan et Shan (*A. biserrata*), a plant of the genus Angelica in the Apiaceae family, is utilized as the traditional Chinese medicine “Duhuo”, demonstrating significant medicinal and industrial value [4,5,6]. Contemporary pharmacological studies have further emphasized the potential benefits of *A. biserrata* roots in detoxification, wound healing, liver-soothing, wind-dispelling, and tranquilizing effects [7]. During the 20th century, numerous scholars documented the various chemical components in *A. biserrata* roots, with coumarins and volatile oil compounds constituting the primary constituents, alongside organic acids and sugars [8,9]. From the perspectives of traditional Chinese medicine pharmacology and plant chemistry, coumarins are considered the most crucial active ingredients [10].

*A. biserrata* roots are extensively distributed, and diverse ecological environments influence their distribution, morphology, physiological effects, and accumulation of secondary metabolites [5,11]. In a previous study utilizing a species distribution model, we found that *A. biserrata* roots exhibit strong ecological adaptability in China, with their suitable distribution area covering 40% of China’s land area. However, this has also resulted in the diversification of *A. biserrata* root varieties and has posed challenges for quality control. Current research on the environmental impacts on *A. biserrata* root quality predominantly focuses on the quantitative disparities in active compounds, whereas the biosynthetic mechanisms and gene expression underlying these variations remain understudied. Furthermore, the suitable growth environment has a significant effect on improving the content and quality of active compounds. Consequently, developing effective analytical methods to comprehensively investigate the ecological adaptation mechanisms of *A. biserrata* roots in various habitats and to identify the key environmental variables influencing these differences is crucial for ecological cultivation and quality control.

In recent years, multi-source data generated by various emerging technologies have been extensively utilized in the comprehensive evaluation of medicinal plant quality. Multi-omics approaches, which enable the deeper exploration of the potential differential characteristics of plants in diverse environments, have been widely studied in the quality evaluation of medicinal plants, thanks to the continuous advancements and cost reductions in high-throughput sequencing technologies [12]. These studies primarily concentrate on identifying molecular alterations in the genome, transcriptome, proteome, and metabolome [13]. Among these, the integrated analysis of transcriptomics and metabolomics not only overcomes the limitations of single-omics methods, but also systematically and comprehensively elucidates the functions and regulatory mechanisms of molecules, so it is more widely used in the quality evaluation of medicinal plants [14,15]. For instance, Bao et al. conducted a quality analysis of *Euryales* Semen originating from different sources and varieties by utilizing untargeted metabolomics [16]. Pathway analysis unveiled the pivotal role of flavonoids in the seed development process of *Euryales* Semen. The research findings suggested a strong similarity in metabolic data among *Euryales* Semen samples from diverse regions. Furthermore, Zhang et al. utilized untargeted metabolomics and transcriptomics to reveal disparities in metabolite accumulation and gene expression between wild and cultivated *ophiocordyceps* sinensis [17]. The integrated analysis of metabolomics and transcriptomics indicated that the genes *IMPDH*, *AK*, *ADSS*, *guaA*, and *GUK* were potentially linked to the synthesis of purine nucleotides and nucleosides, providing a fresh perspective on the molecular underpinnings of metabolic variations in medicinal fungi.

With the continuous progress of computer algorithms, the combination of multi-source data and machine learning has become an important method to evaluate the influence of various environmental factors on the quality of medicinal plants [18]. Machine learning enables the assessment of feature importance for prediction outcomes, thereby enhancing the identification of key factors in datasets. This capability is particularly valuable for high-dimensional data, as it facilitates the elimination of irrelevant features, simplifies the model architecture, and improves interpretability [19,20]. For instance, Liu et al. applied machine learning to analyze the correlation between climate data and *Panax notoginseng* saponin content, revealing that saponin levels were negatively correlated with annual average temperature and annual temperature range. Lower annual average temperatures and reduced annual temperature ranges were shown to promote saponin accumulation [21].

Beyond identifying key environmental variables, deep learning within machine learning has been progressively applied to predict optimal environments due to its advantages in capturing nonlinear relationships between phenotypic traits and yields. For instance, Gharghory et al. proposed an enhanced architecture based on LSTM recurrent neural networks to forecast greenhouse microclimates [22]. Shi et al. integrated deep learning with the Sparrow search algorithm to predict greenhouse microclimates, thereby improving seedling environmental adaptability [23]. Among the various algorithms, deep neural network models (DNNs) are favored for their proficiency in processing large-scale data and time-series predictions [24]. While DNNs excel at capturing spatial features, their lack of explicit memory mechanisms for temporal dependencies renders them relatively weak in time-series modeling. Consequently, optimization algorithms play a pivotal role in enhancing model performance [25]. Commonly employed optimization methods include Particle Swarm Optimization (PSO), Genetic Algorithm (GA), and Bayesian Optimization (BO), among others [26]. Compared to PSO and GA, BO demonstrates superior global exploration capabilities [27]. By iteratively selecting the most promising parameter combinations for evaluation, BO effectively reduces the search space and enhances the optimization efficiency [28]. Cho et al. also analyzed four baseline strategies for DNN hyperparameter optimization in their study, revealing that BO consistently delivered top-tier or near-top-tier performance across all DNN benchmark tests [29].

Based on the aforementioned background, this study investigated *A. biserrata* roots from different habitats, employing transcriptomics and metabolomics to conduct in-depth analyses of variations in secondary metabolites and genetic information across habitats. Integrated with machine learning, we identified the key environmental variables driving these differences. Additionally, a quantitative model linking growth environments to bioactive molecules was established using deep learning and Bayesian Optimization. This research aimed to elucidate the biological characteristics and differential mechanisms of *A. biserrata* roots under diverse habitats, thereby providing a scientific basis and theoretical guidance for advancing studies on the interactions between medicinal plants and their ecological environments.

## 2. Results

### 2.1. Metabolic Differences and Similarities of Ecotype Samples

Through the quality control of internal standards and QC samples, the pretreatment, derivatization, sample loading, and mass spectrometry system stability of the experimental parts were analyzed and evaluated. As depicted in Figure S1, the PCA model graph obtained through 7-fold cross-validation shows that the QC samples cluster closely together, indicating good stability and reproducibility in this experiment. In this study, a total of 4982 metabolites were identified and analyzed based on The Human Metabolome Database (HMDB), Lipidmaps (v2.3), and the METLIN database. Among them, the 995 secondary metabolites mainly included coumarins and their derivatives, flavonoids, prenol lipids, and steroids and steroid derivatives (Table 1), of which prenol lipids accounted for the largest proportion. The prenol lipids in *A. biserrata* roots were mainly terpenoids and volatile oils.

**Figure 1:**
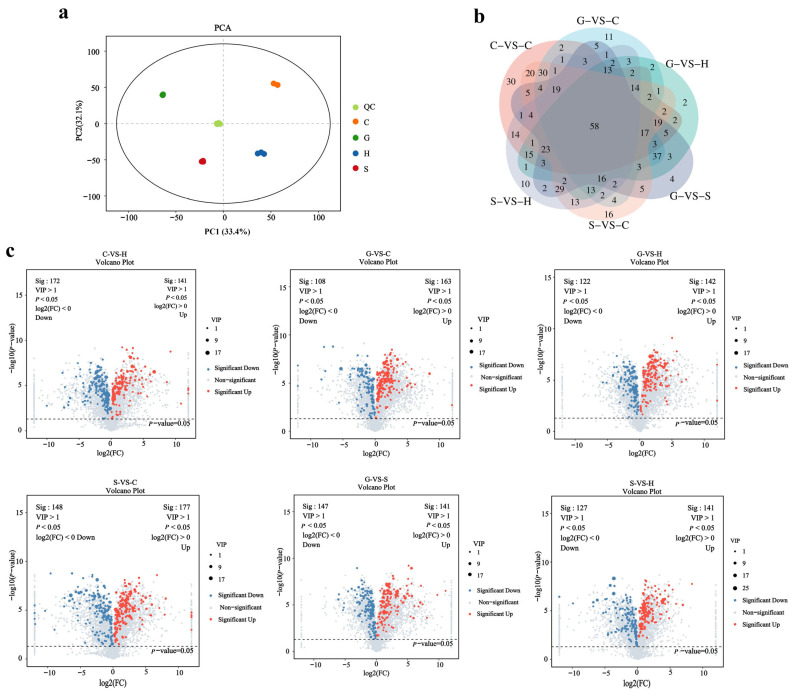
Metabolomics profiles of Ecotype Samples. (**a**) PCA score plot; (**b**) Venn plot; (**c**) Volcano plot.

To evaluate the diversity of secondary metabolites among the four ecotype samples, principal component analysis (PCA) was employed. PCA represents an unsupervised pattern recognition technique frequently utilized to visualize overarching clustering patterns among distinct groups and to assess the variability within the same set of samples [30]. The PCA results indicated that the biological replicates of the four samples clustered together in distinct regions, highlighting significant differences. PC1 and PC2 contributed 35.2% and 33.4%, respectively, to the sample separation (Figure 1a). To investigate the influence of different habitats on the metabolites of *A. biserrata* roots, an OPLS-DA model was established to discern the metabolic distinctions between each pair of the four distinct habitats. Based on the high predictability (Q2) of the OPLS-DA models, coupled with permutation tests for additional verification (Figure S2), models exhibiting high predictiveness and reliability were developed. As illustrated in Figure S3, the score plots of the six models exhibit a clear distinction between the various groups.

Through screening for differential metabolites, it was found that the differential metabolites (DAMs) among different varieties were primarily characterized by variations in their metabolite content, rather than their types. The screening results were visualized using Venn diagrams (Figure 1b) and a Volcano plot (Figure 1c). The Volcano plot reveals 313 DAMs between C and G (141 upregulated, 172 downregulated), 266 DAMs between C and H (142 upregulated, 122 downregulated), 286 DAMs between S and H (141 upregulated, 127 downregulated), 271 DAMs between G and C (163 upregulated, 108 downregulated), 325 DAMs between S and C (177 upregulated, 148 downregulated), and 288 DAMs between G and S (141 upregulated, 147 downregulated). These differential metabolites, representing a significant proportion of the organooxygen compounds (13.8–18.8%), prenol lipids (8.6–11.3%), coumarins and their derivatives (7.9–11.3%), carboxylic acids and their derivatives (7.9–10.3%), fatty acyls (8.9–11.7%), and flavonoids (5.9–8.9%), are detailed in Table 2. Among these, organooxygen compounds constituted the highest proportion in all comparison groups. The differences in secondary metabolites were primarily attributed to coumarin and its derivatives, as well as flavonoids. Furthermore, based on the Venn diagram, 58 shared DAMs were identified through pairwise comparisons, with cluster analysis indicating the predominant occurrence of coumarins and organic oxygen compounds, with coumarins notably comprising 22% (Figure S4).

KEGG enrichment analysis was conducted on the differential metabolites of each control group: C-VS-H, G-VS-C, G-VS-H, G-VS-S, S-VS-C, and S-VS-H, which were enriched in 51, 76, 49, 62, 63, and 62 pathways, respectively (Tables S3 and S4). The top 20 pathways with the lowest *p*-values were selected to construct a circular plot for KEGG enrichment analysis (Figure S5). The results indicated that the enriched pathways across all control groups primarily belonged to the categories of metabolism, environmental information processing, organismal systems, and human diseases. Among these categories, metabolism was the most enriched. In each control group, the most enriched differential metabolites were related to the biosynthesis of various plant secondary metabolites, followed by amino acid metabolism. Furthermore, the metabolites enriched in the biosynthesis pathways of secondary metabolites were primarily coumarins and derivatives.

### 2.2. Genetic Information Differences and Similarities of Ecotype Samples

This study generated a total of 81.24 Gb of clean data based on transcriptome sequencing, with an effective data volume ranging from 5.97 to 7.01 Gb per sample. The Q30 base distribution ranged from 93.81% to 94.67%, with an average GC content of 42.5% (Table S5). The length distribution of the annotated genes and the sample FPKM expression distribution range are shown in Figure S6a,c. Based on the PCA (Figure S6b), the biological replicates of the four samples exhibited significant differences across distinct regions. The clustering analysis of the sequencing samples demonstrated tight clustering of the three biological replicates in each group, indicating the high reproducibility of the transcriptome data (Figure S6d).

**Figure 2:**
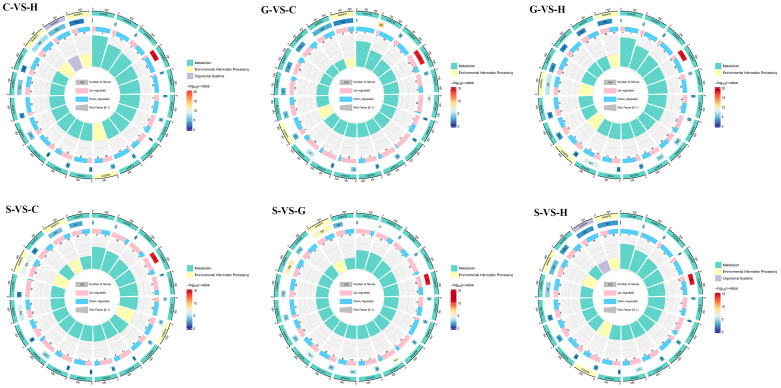
KEGG enrichment circle diagrams of DEGs. First circle: Enriched classifications, with the outer circle representing the coordinate scale for the number of genes. Different colors signify different classifications. Second circle: The number of genes in that classification within the background set, along with the *q*-value or *p*-value. The longer the bar, the more genes are present; the redder the color, the smaller the value; the bluer, the larger. Third circle: Bar chart showing the proportion of up- and downregulated genes. Light red represents the proportion of upregulated genes, while light blue represents the proportion of downregulated genes. The specific numerical values are displayed below. Fourth circle: RichFactor values for each classification (the number of foreground genes in the classification divided by the number of background genes). The background grid lines indicate increments of 0.2.

The numbers of differentially expressed genes (DEGs) identified in the differential comparisons of C-VS-H, G-VS-C, G-VS-H, G-VS-S, S-VS-C, and S-VS-H were 22,451, 15,782, 21,967, 21,503, 18,357, and 19,590, respectively, as illustrated in Figure S7. The expression profiles of the living genes in four different habitats were analyzed, and the results indicated that the types were essentially the same. GO enrichment analysis was employed to describe the functions of the DEGs in *A. biserrata* root samples from four distinct habitats. All DEGs were effectively annotated into the three functional categories of “cellular component”, “molecular function”, and “biological process” in the GO analysis. The top 30 enriched GO terms were shown in Figure S8. Through pairwise comparisons, it was observed that within the biological process category, the majority of DEGs were enriched in DNA integration and DNA recombination. With respect to the cellular component category, the DEGs exhibited the highest enrichment in the integral component of the membrane and the nucleus. In the category of molecular function, the DEGs were predominantly enriched in metal-ion-binding and ATP-binding activities. KEGG enrichment pathway analysis was performed on the DEGs, and all were successfully annotated into categories including “genetic information processing”, “metabolism”, “cellular processes”, and “environmental information processing” (Table S6). The 20 pathways with the lowest *p*-values were chosen to construct circular plots for the KEGG enrichment analysis (Figure 2). Notably, within the “metabolism” category, phenylpropanoid biosynthesis exhibited the smallest *p*-values among all comparison groups and demonstrated the highest enrichment based on the number of genes.

To further explore the overall differences in genes, we annotated them to transcription factors. A total of 1691 Unigenes were annotated as transcription factors, among which AP2/ERF-ERF, NAC, and WRKY were the transcription factors with a relatively large number of genes, with 123, 121, and 106 genes, respectively. Upon comparing the distribution of all Unigenes and differentially expressed Unigenes (upregulated and downregulated) in terms of their transcription factor profiles, it was observed that in each comparison group, the transcription factor with the highest number of differentially expressed genes was AP2/ERF-ERF. Specifically, in C-VS-H, there were 17 upregulated and 47 downregulated; in G-VS-C, 28 upregulated and 14 downregulated; in G-VS-H, 27 upregulated and 44 downregulated; in S-VS-C, 44 upregulated and 16 downregulated; in S-VS-G, 32 upregulated and 15 downregulated; and in S-VS-H, 28 upregulated and 34 downregulated (Figure S9).

### 2.3. Comprehensive Analysis of Metabolomics and Transcriptomics

To investigate the relationship between DEGs and DAMs in *A. biserrata* roots from different habitats, we conducted a comprehensive analysis. Spearman correlation coefficients (PCCs) were calculated among the top 30 DEGs and DAMs, and the concentration of the data was visually analyzed. As shown in Figure S10A, the results indicated that in pairwise comparisons across the four distinct habitats, most DAMs were significantly correlated, either positively or negatively, with DEGs. The data comparison revealed that among all the comparison groups, the proportion of metabolites enriched in the synthesis pathway of secondary metabolites was the highest, and these secondary metabolites all belonged to the coumarin class in phenylpropanoid. Co-enrichment pathway analysis was further conducted on DAMs and DEGs, visualizing the top 30 pathways (Figure S10B). Many pathways related to carbohydrates and amino acids exhibited significant differences, including carbohydrate metabolism, such as pentose and glucuronate interconversions; starch and sucrose metabolism; galactose metabolism; and amino acid metabolism, with significant pathways like cyanoamino acid metabolism and histidine metabolism.

### 2.4. Analysis of the Synthetic Pathway of the Main Active Compound: Coumarins

**Figure 3:**
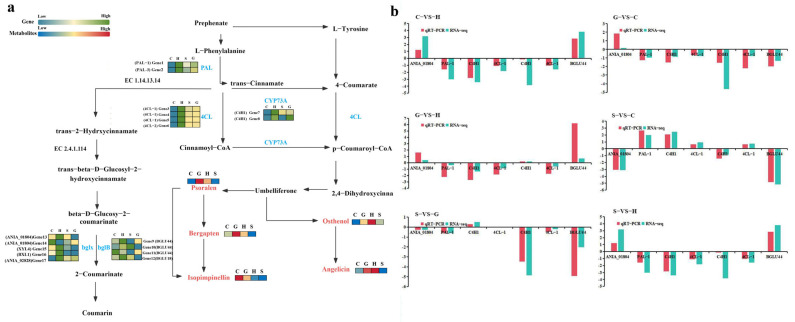
(**a**) Gene changes in metabolites and regulatory enzymes in the process of coumarin metabolism; (**b**) quantitative real-time PCR (qRT-PCR) validation of select genes.

From the perspective of the synthetic pathway, it was mainly the upstream gene that regulated the downstream coumarin difference (Figure 3a). These genes included *PAL-1* and *PAL-2* which were associated with the synthesis of trans-cinnamate from L-phenylalanine; *BGLU44*, *ANIA_01804*, *XYL4*, *BXL1*, *BGLU18*, and *ANIA_02828* with the synthesis of cis-2-Hydroxycinnamate from beta-D-Glucosyl-2-coumarinate; and *C4H1*, *4CL-1*, and *4CL-2* with the synthesis of various coumarins and their derivatives from trans-cinnamate.

In order to verify the reliability of the transcriptome data, we used qRT-PCR to determine the expression levels of seven genes involved in coumarins’ metabolism (Figure 3b). The expression patterns of most genes in all comparison groups were similar to those obtained in the RNA-Seq analysis. Therefore, the results of the RNA-seq analysis that we completed have high reproducibility and reliability, which is helpful to further study the key genes of coumarin accumulation in *A. biserrata* roots. In addition, the RNA-Seq and qRT-PCR results showed that the data could evaluate the upregulation and downregulation of gene expression. Comparative analysis revealed fifteen downregulated and two upregulated genes in the C-VS-H comparison group. Through pairwise comparison analysis, it was observed that in the C-VS-H comparison group, fifteen genes were downregulated and two were upregulated. In the G-VS-C group, seven genes were upregulated and ten were downregulated. For G-VS-H, fifteen genes were downregulated and two were upregulated. In S-VS-C, five genes were upregulated and twelve were downregulated. In S-VS-G, eight genes were upregulated and nine were downregulated. Finally, in S-VS-H, fourteen genes were upregulated and three were downregulated (Table 3). The differential changes in metabolites were primarily observed in derivatives of the secondary metabolite coumarin, such as osthenol and psoralen.

### 2.5. Interaction Between Environment and Global Transcriptome as Well as Metabolome

**Figure 4:**
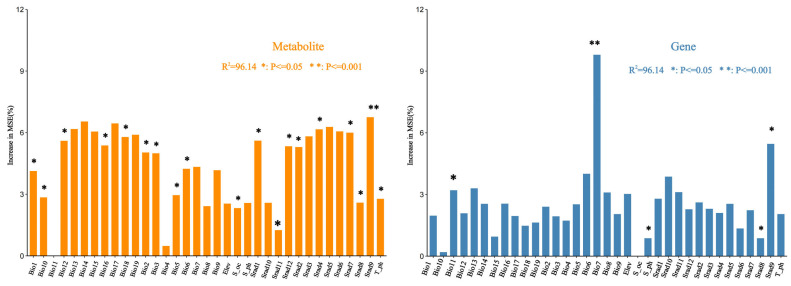
RF of environmental variables and transcriptomics and metabolomics.

This study employed R language to conduct Random Forest (RF) analyses on 38 environmental variables and the PC1 feature axes derived from two omics datasets. As shown in Figure 4, most of the environmental variables were significant for the ranking of the importance of the metabolomics data. This may be attributed to the intricate nature of transcriptomics data, and the genes may have showed diversity under the action of the environment. Further analysis showed that the most important and significant environmental variable for the ranking of metabolomics data was the solar radiation in September, and the rankings of the precipitation factor (Bio12–Bio19) and solar radiation factor (Srad1-12) were more important than that of the temperature factor (Bio1–11). The environmental variables temperature annual range (Bio7) and solar radiation in September were the most important for the transcriptome of effects.

### 2.6. Interaction and Interaction Network Between Environment, Coumarin Metabolites, and Genes

**Figure 5:**
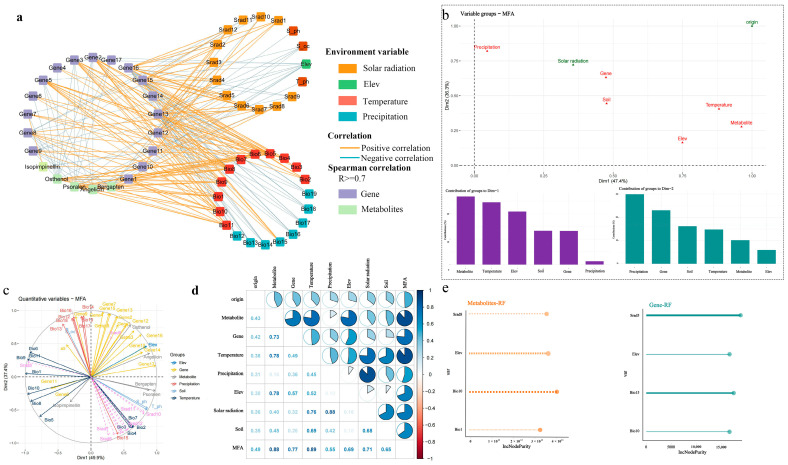
(**a**) Environment–metabolite–gene correlation diagram; (**b**) MFA variable set contribution graph; (**c**) contribution of 20 variables to Dim1 and Dim2 rankings; (**d**) Spearman correlation analysis of different datasets; (**e**) random forest feature sorting.

Through the interaction network diagram, it was found that osthenol has a significant negative correlation with Bio1, Bio5, Bio6, Bio8, Bio9, Bio10, and Bio11, and a positive correlation with Elev (Figure 5a). The relationship between environmental factors and coumarin metabolites, as well as the genes related to their synthesis, was quite complex, making it difficult to assess the key environmental variables that influenced coumarin differences (Figure S11). Therefore, this study further evaluated the key environmental variables affecting these differences using machine learning and statistical analysis methods.

In this study, the ‘FactoMineR’ package of R language was used for MFA, and the differential coumarin metabolites, their related transcriptome gene expression, environmental factors, and origin information were organized into eight datasets, in which the environmental factors were divided into five categories (Figure S12). By balancing the influence of each group of variables, the comprehensive relationship between each group of variables was analyzed. According to the position of the variable set in the ranking graph, the correlation and relative contribution between the variable set and the MFA feature group could be evaluated. The results showed that the metabolites contributed the most to Dim1, followed by the environmental variable temperature, and the environmental variable rainfall contributed the most to Dim2 (Figure 5b). Figure 5c shows the contribution of the first 20 variables to the ranking of Dim1 and Dim2. Among them, the environmental factor altitude had the highest contribution to Dim1, and the soil factor (S_oc) had the highest contribution to Dim2. Spearman correlation analysis of different datasets found that metabolites were significantly positively correlated with altitude, genes, and altitude, and that genes had the highest correlation with altitude (Figure 5d).

Considering the comprehensiveness and complexity of ecological factors, we used R V4.3.1 software to calculate the Spearman correlation coefficient of the environmental variables. Combined with the results of MFA, the variables with correlation coefficients exceeding |0.8| only retained the variables with the highest contribution, and finally, 11 environmental variables were selected. The ‘vegan’ package in R language was used to perform RDA on 11 environmental variables related to coumarin-related differential metabolites and differential key genes. The results showed that the four environmental variables with the highest degree of interpretation and significance with genes were Elev, Bio10, Srad5, and Bio15. The four environmental variables with the highest degree of interpretation and significance with metabolites were Srad8, Bio10, Elev, bio1 (Figure S13a). VPA was used to evaluate the explanatory ratio of environmental factors to gene and metabolite changes. The results showed that elev had the highest explanatory ratio of 16.51% for metabolites, followed by bio10 with 5.88%. In terms of genes, Srad5 had the highest interpretation ratio of 51.08%, followed by bio15 of 50.50% (Figure S13b). The cross-validation of the random forest analysis of environmental variables showed that the selection of two important variables from the four environmental variables would obtain the ideal regression results, and the error was minimized. The ranking of importance results showed that elev and bio10 had the most important effect on the content of coumarin metabolites, and srad5 and bio15 were the most important for the expression of genes related to coumarin synthesis, which was consistent with the results of VPA (Figure 5e). The marginal effects of important environmental variables on metabolites and gene expression can be seen in the partial dependence plot. If the variable has little effect on the result, the partial dependence plot should be a horizontal line. It can be seen from the diagram that the effects of coumarin metabolites and gene expression under different environmental variables are significantly different (Figure S14).

### 2.7. Optimal Environment for Active Compound Accumulation

**Figure 6:**
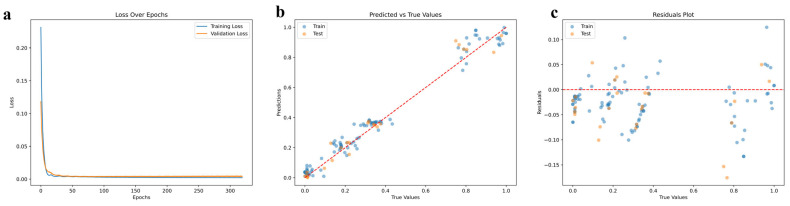
(**a**) Loss over epochs; (**b**) relationship between true and predicted values; (**c**) residual plot.

This study employed Bayesian Optimization in conjunction with deep neural networks to identify the optimal environmental conditions for the active compounds in *A. biserrata* roots, thereby offering a valuable reference for the ecological cultivation of Chinese medicinal herbs. The MSE for the test set was 0.0043, with a training value of 0.0025. The RMSE for the test set was 0.0654, with a training value of 0.0495. Additionally, the MAE for the test set was 0.05, with a training value of 0.0388. The R^2^ value was 0.9772, which was close to 1 (Table 4). Figure 6a illustrates the change in the loss function over training epochs, exhibiting a general trend of an initial decrease followed by stabilization. Figure 6b presents the relationship between the actual and predicted values, where all data points lie approximately along a straight line. Figure 6c is a residual plot that demonstrates that the residuals are randomly distributed around zero, with no discernible patterns or trends observed. Specifically, the optimal values obtained were as follows: Bio1 of 9.9878302 °C, Bio12 of 1493 mm, Elev of 1728 m, SradSUM of 152,643 kJ∙m^−2^∙day^−1^, and S_oc (soil organic carbon) content of 11,883.

## 3. Discussion

### 3.1. Differences Between Metabolites and Heritage Information of Ecotype A. biserrata Roots

A comparison of information on metabolite and gene expression differences between the four ecotype samples revealed that the differences were only in terms of content and did not differ between species, suggesting that the different habitat environments only altered the differences in content. KEGG enrichment analysis, based on the screened differential metabolites and genes, revealed that the most abundant and significant differential metabolite enrichment pathway was secondary metabolite synthesis. Of particular note was the observation that all of these secondary metabolites were coumarin analogs. The most abundant and significant differential gene enrichment pathway was found to be phenylpropanoid synthesis. In many medicinal plants, the phenylpropanoid pathway serves as a pivotal biosynthetic route for critical secondary metabolites, such as coumarins. These metabolites, derived from the phenylpropanoid pathway, not only act as indicators of plant responses to environmental stressors but also function as key mediators in plant pathogen resistance [31]. Studies have demonstrated that variations in growth environments significantly influence the types, concentrations, and biosynthetic pathways of secondary metabolites in medicinal plants [32]. As essential bioactive compounds and adaptive products to environmental conditions, the synthesis and accumulation of secondary metabolites in medicinal plants are closely associated with ecological factors [33]. For example, Du et al. reported that the differential metabolites in *Eucommia ulmoides* cultivated across distinct regions predominantly originated from flavonoid compounds produced via the phenylpropanoid pathway, with flavonoids constituting its primary bioactive constituents [34]. Similarly, Zhang et al. revealed that environmental disparities in the growth habitats of *F. dibotrys* were primarily elucidated by variations in the abundance of phenolic and flavonoid compounds [35].

In the present study, the biosynthetic process of coumarin was illuminated through an integrated analysis of transcriptomics and metabolomics. The results obtained demonstrated significant disparities between the upstream gene and the downstream coumarin metabolites. This observation suggested the possibility of regulatory influence by the upstream gene on the downstream metabolic differences. Dong et al. demonstrated that in Angelica sinensis (a species of the genus Angelica), when the expression levels of genes related to the biosynthesis of its active compounds—ferulic acid (*PAL1*, *4CLL4*, *4CLL9*, *C3H*, *HCT*, *CCOAOMT*, and *CCR*) and flavonoids (*CHS* and *CHI*)—were increased under varying temperatures, the concentrations of these metabolites were simultaneously elevated [36]. This further indicated that environmental factors regulated the accumulation of downstream metabolites through upstream genes. The upstream genes included *PAL-1*, *PAL-2*, *BGLU44*, *ANIA_01804*, *XYL4*, *BXL1*, *ANIA_02828*, *C4H1*, *4CL-1*, *F6H1-3*, and *4CL-2*. Han et al. reported that the biosynthetic pathway of simple coumarins involved 10 gene families, comprising *C4H*, *C2’H*, *C3H*, *4CL*, *C3’H*, *CCoAOMT*, *COMT*, *COSY*, *F6’H*, and *HCT* genes. Most annotated gene fragments in this study were classified into these gene families [37]. To validate the reliability of the transcriptome data, we employed qRT-PCR to determine the expression levels of seven genes involved in coumarin metabolism. The expression patterns of most genes across all control groups showed consistency with those obtained from RNA-Seq analysis. Consequently, our RNA-Seq results demonstrated high reproducibility and reliability, providing valuable insights into the coumarin biosynthetic pathway.

Through an analysis of the expression levels of coumarin metabolites in *A. biserrata* roots from different regions, we found that the coumarin content in samples from Hubei was significantly higher than that from other regions, whereas the content in samples from Shaanxi was the lowest among all. Han et al. revealed that there were considerable variations in the mass fractions of indicator components in *A. biserrata* roots from different regions, ranked, from high to low, as Wushan, Hubei, Wuxi, Sichuan, Shaanxi, and Gansu [38]. The results of this study were largely consistent with theirs, suggesting that regions such as Hubei and the Chuan Yu area (Sichuan–Chongqing) possess a more sensitive environmental response mechanism, which facilitates the accumulation of coumarin components in *A. biserrata* roots.

### 3.2. Key Environmental Variables Affecting A. biserrata Root Active Substance Accumulation and Gene Expression

Environmental factors exert a significant impact on the synthesis and gene expression of plant secondary metabolites [39]. In this study, environmental variables were found to have some effect on the overall metabolite and transcriptional PC1 feature axes. However, it was not possible to identify the key environmental variables. This may be attributed to the complexity of the entire dataset and the interactions among environmental variables. Previous studies have demonstrated that environmental variables are complex and dynamic, and their impact on the accumulation of plant metabolites constitutes a comprehensive process [40]. In nature, the plant stress response represents a highly coordinated signaling event that has evolved over thousands of years [41]. The influence of environmental factors on plants is never mediated solely by a single factor [34]. Climate change will induce metabolic alterations in plants and modify the accumulation patterns of primary and secondary metabolites [42]. For instance, barley grown under a single stress factor can adapt and enhance its resistance, whereas the combination of climatic factors mitigates this effect [43]. Likewise, tomato (*Solanum lycopersicum*) plants exhibit superior performance when grown at elevated temperatures and under higher light conditions compared to when grown under standard conditions [44].

By utilizing machine learning, statistical analysis, and constructing interaction networks, we further analyzed the key environmental variables influencing the differences in coumarin metabolites and gene expression. Our final analysis revealed that elev (elevation) and bio10 (mean temperature of the warmest quarter) were key variables influencing differences in coumarin metabolites, whereas srad5 (solar radiation in May) and bio15 (precipitation seasonality) were crucial for gene expression. Altitude served as the primary regulatory factor influencing growth, development, and the accumulation of active substances [45]. Prior research has demonstrated that altitude exerts a significant influence on terpenoids, phenylpropanoids, fatty acid biosynthesis, and flavonoid biosynthesis [46,47,48,49]. Optimal altitude conditions impact quality by altering the distribution pattern of photosynthetic products and the rate of dry matter accumulation [50].

The radiation in May (annual sunshine hours) had a significant effect on the expression of genes associated with coumarin synthesis in *A. biserrata* roots, which could be attributed to May being the flowering period for *A. biserrata* roots [51]. Elevational gradients led to a substantial increase in ultraviolet (UV) radiation intensity, particularly within the UV-A and UV-B spectral bands, while coumarin biosynthesis exhibited pronounced sensitivity to UV exposure. Research indicates that variations in UV-A and UV-B radiation activate the expression of genes encoding key enzymes in phenylpropanoid biosynthesis [52]. Escobar et al. demonstrated that prolonged UV-B exposure triggered divergent expression patterns of phenylpropanoid biosynthetic genes between two *Vaccinium corymbosum* cultivars, accompanied by distinct disparities in total phenolic compounds and flavonoid accumulation [53]. Similarly, Lei et al. revealed that high-altitude adaptation in *Draba oreades Schrenk* enhanced resilience to intensified UV radiation and concurrent low-temperature stress, thereby driving dynamic shifts in phenylpropanoid and flavonoid metabolite profiles [54].

### 3.3. Deep Learning Predicts the Optimal Suitable Environment for Active Substances of A. biserrata Roots

The active compounds in medicinal plants constitute the cornerstone of their therapeutic efficacy, and the environment exerts a substantial influence on the content and quality of these compounds. Optimizing the growth conditions of medicinal plants can augment the concentration and quality of their bioactive compounds. In this study, a combination of Bayesian Optimization and deep neural networks was employed to simulate and predict the optimal environment for the bioactive compounds of *A. biserrata* roots. The results indicated that the model’s evaluation metrics, including MSE, RMSE, MAE, and R^2^, exhibited excellent performance. Notably, the R^2^ value approached 1, signifying an exceptional fit of the model to the data, which nearly fully explained the variance therein. Concurrently, the gradual decrease in the loss function across training epochs indicated that the model was learning and enhancing its predictive capabilities. Ultimately, its stabilization suggested that the model had converged to the optimal solution [55]. The high consistency between the actual and predicted values demonstrated the model’s accuracy in predicting the growth environment of *A. biserrata* roots. The random distribution of residuals, devoid of any discernible patterns or trends, typically indicates that the model is appropriate and free from significant systematic errors. Specifically, the predicted optimal growth conditions for the bioactive compounds of *A. biserrata* roots were as follows: Bio1 (annual mean temperature) of 9.9878302 °C, Bio12 (annual precipitation) of 1493 mm, Elev (elevation) of 1728 m, SradSUM (total solar radiation) of 152,643 kJ∙m^−2^∙day^−1^, and S_oc (soil organic carbon) of 11,883.

The relevant literature indicates that *A. biserrata* roots thrive in cool, humid environments, are cold-hardy, and have an optimal growth temperature range of 15–25 °C [56]. However, some studies suggest more vigorous growth at 8–12 °C. Although the simulated temperature values are slightly below the optimal range reported in the literature, they remain within the growth range of *A. biserrata* roots. Lower temperatures reduce the growth rate while potentially inducing environmental stress signals, which activate secondary metabolic pathways, thereby enhancing the accumulation of secondary metabolites [57]. *A. biserrata* roots adapt to a wide range of annual precipitation, from 600 to 1500 mm. High precipitation maintains soil moisture, fostering growth and active compound accumulation. *A. biserrata* roots are predominantly found in alpine regions, at altitudes of 1000–2600 m [58]. The predicted altitude aligns with its growth range. High-altitude conditions, including lower temperatures, higher humidity, and fertile soil, favor *A. biserrata* roots’ growth. Although the literature does not directly address total solar radiation, *A. biserrata* roots’ shade-loving nature suggests that excessive radiation may be harmful. However, moderate radiation is crucial for photosynthesis. Soil organic carbon is a crucial indicator for assessing soil fertility. *A. biserrata* roots thrive in fertile, loose soil rich in organic matter [59]. According to relevant research findings, the soil organic carbon content value predicted in this study is highly beneficial for the growth of *A. biserrata* roots, promoting root development and nutrient absorption, thereby facilitating growth and the accumulation of active compounds. In summary, the results of this study are generally consistent with the actual situation of high-quality *A. biserrata* root cultivation, indirectly proving the accuracy and precision of the model constructed in this study. Of course, actual cultivation still requires the fine-tuning of the planting area in conjunction with local conditions and the growth habits of *A. biserrata* roots to improve its yield and quality.

## 4. Materials and Methods

### 4.1. Preparation of A. biserrata Root Materials

**Figure 7:**
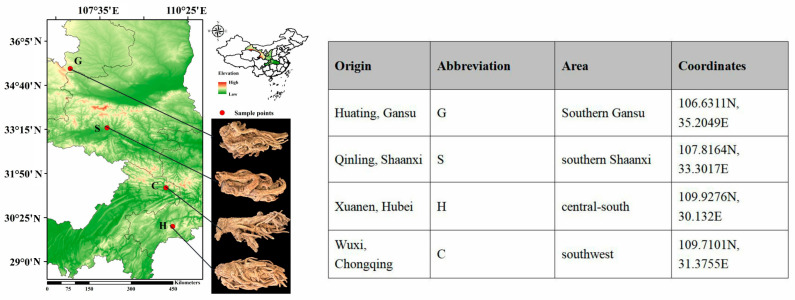
Collection point information and sample images.

Fresh, high-quality samples of *A. biserrata* roots from four distinct regions were selected, without the utilization of genetically modified organisms (GMOs) or their derivatives, nor the application of chemically synthesized insecticides, fertilizers, growth regulators, or other chemical substances. Farmhouse manure was applied as the standard practice, with a harvesting cycle of two years, and the harvest occurred in October. The collection sites were located away from urban areas, industrial and mining zones, sources of industrial pollution, and domestic waste disposal facilities, encompassing the primary areas of artificial cultivation for *A. biserrata* roots. At each sampling site, three replicate root samples were randomly collected, placed in sterile plastic bags, maintained on ice, and transported immediately to the laboratory. The harvested roots were rinsed with distilled water to remove surface contaminants and subsequently blot-dried with filter paper. All samples were flash-frozen in liquid nitrogen and stored at −80 °C to ensure the preservation of biomolecular integrity for subsequent transcriptomic and metabolomic analyses. The details of the collection sites are presented in Figure 7.

### 4.2. Transcriptome Analysis

This study utilized Illumina NovaSeq 2000 sequencing technology to explore the variations in the gene expression of *A. biserrata* roots across diverse habitats. After annotating the Unigenes, the sequence reads were aligned to these Unigenes by utilizing the Bowtie2 V2.5.2 software. Subsequently, the expression levels of the Unigenes were quantified using the express software, yielding FPKM values [60]. Differential expression multiples were computed using DESeq2 V3.11 software, and differential expression analysis was conducted by employing the negative binomial distribution (NB) test. The criteria adopted for identifying differentially expressed genes were a *q*-value of less than 0.05 and a FoldChange greater than 2. The Unigenes were mapped onto the Kyoto Encyclopedia of Genes and Genomes (KEGG) to annotate their biological pathways. Gene Ontology (GO) term assignment was performed by aligning the Unigenes with entries in the Swiss-Prot database and with the corresponding GO terms [61]. For detailed procedures and methods, please refer to the Supplementary Materials.

### 4.3. Metabolite Determination and Analysis

This study utilized liquid chromatography–tandem mass spectrometry (LC-MS/MS) to quantify differential metabolite profiles. A combination of multidimensional and unidimensional analytical approaches was employed to screen for differentially expressed metabolites between groups. In the OPLS-DA and PLS-DA analyses, Variable Importance in Projection (VIP) scores were used to evaluate the influence and explanatory power of metabolite expression patterns in distinguishing between sample groups. This aided in the identification of biologically significant differential metabolites. Subsequent validation using *T*-tests was conducted to determine the statistical significance of the differentially expressed metabolites between groups. The selection criteria were a *p*-value less than 0.05 and a VIP score greater than 1. The identified differential metabolites were subsequently analyzed for metabolic pathway enrichment using the KEGG database V114.0 [34,62].

### 4.4. Quantitative Real-Time Polymerase Chain Reaction (qRT-PCR)

To validate the RNA-seq data, eight differentially expressed genes (DEGs) involved in coumarin metabolism were selected for quantitative real-time PCR (qRT-PCR) analysis. The primer sequences were designed in-house and synthesized by Qingke Biotech (Beijing Tsingke Biotech Co., Ltd., Beijing, China) based on the mRNA sequences retrieved from the National Center for Biotechnology Information (NCBI) database, as detailed in Table S1. The Actin (ACT) gene served as a reference control, with the forward primer sequence of TGGTATTGTGCTGGATTCTGGT and the reverse primer sequence of TGGATCACCACCAGCAAGG producing an amplicon size of 10^9^ base pairs (bp). Relative changes in gene expression levels were then determined using the 2^−ΔΔCt^ method [63].

### 4.5. Environment Variable

Given the comprehensive and complex nature of ecological factors, we selected 38 environmental variables to explore the mechanism by which the environment influenced the accumulation of metabolites in *A. biserrata* roots [64] (Table S2). These variables encompassed climate data, soil data, and terrain data. The climate data consisted of solar radiation and 19 bioclimatic factors, collected monthly from January to December. The data were sourced from the WorldClim V2.1 database (http://www.worldclim.org, accessed on 15 October 2023), with a spatial resolution of 2.5 km × 2.5 km. Six soil factors, including soil types and various physical and chemical properties, were selected from the World Soil Database (https://www.fao.org/home/en/, accessed on 15 October 2023) with a spatial resolution of 30 m. Using ArcGIS 10.8, environmental variable values from the 38 factors were extracted to the specific sampling points to obtain relevant information on the environmental conditions.

### 4.6. Deep Learning and Statistical Analysis

Based on R software version 4.3.1, we utilized two machine learning approaches, namely random forest and Multiple Factor Analysis (MFA), in conjunction with Redundancy Analysis (RDA), Spearman correlation analysis, and Variance Partitioning Analysis (VPA), to identify the pivotal environmental variables that contribute to the significant disparities in active substances and their corresponding gene expression. The environment–genetic–metabolite network was visualized utilizing R software version 4.3.1 in conjunction with Cytoscape version 3.5 [65].

To optimize the optimal cultivation environment, we concentrated on maximizing the content of osthole, a representative coumarin derived from the roots of Ap, employing DNNs coupled with Bayesian Optimization for predictive purposes. The DNNs, in conjunction with Bayesian Optimization, was implemented using Python 3.12, with Keras and scikit-optimize employed for the construction, training, and evaluation of the neural network. The dataset was randomly split into three subsets: training, validation, and testing. During the neural network training process, 80% of the data were used for training, 10% for validation, and 10% for testing. The transfer functions for the hidden and output layers were the hyperbolic tangent sigmoid function (tansig) and the linear function (in), respectively. Throughout the construction process, we ensured the data quality by verifying the absence of missing values, converting pertinent dataset columns to floating-point types to facilitate mathematical calculations, and augmenting the dataset by replicating the original data and introducing random perturbations to enhance data diversity and model generalization. The data were scaled to the [0, 1] range utilizing MinMaxScaler V1.6.0, a widely used data preprocessing technique for deep learning models that aids in accelerating model convergence [66]. BayesSearchCV V0.8.1 was employed for the Bayesian Optimization of hyperparameters.

The procedure was conducted 300 times to assess the model’s accuracy, employing metrics such as Mean Squared Error (MSE), Root Mean Squared Error (RMSE), Mean Absolute Error (MAE), and the Coefficient of Determination (R^2^). Additionally, visual aids, including plots of the predicted versus actual values, residual plots, and graphs depicting the variation in the loss function across training epochs, were utilized. To evaluate the suitability of the overall environment, five key environmental variables were selected: Bio1 (annual mean temperature), Bio12 (annual precipitation), Elev (elevation), SradSUM (total solar radiation), and S_oc (soil organic carbon). These variables were employed to predict the optimal environmental conditions.

## 5. Conclusions

This study aimed to address the challenges posed by the extensive distribution and environmental variability of medicinal plants in achieving standardized quality control and maintaining genetic diversity. Using the roots of the widely distributed *A. biserrata* roots as a model, we integrated multi-omics approaches, computational ecology, and environmental big data to investigate the mechanisms by which ecological factors regulate their bioactive molecules. The results demonstrated that environmental variables significantly influenced both plant growth and the accumulation of bioactive compounds, with the coumarin biosynthesis pathway exhibiting the most pronounced metabolic divergence. By reconstructing the coumarin biosynthetic pathway, we identified the upstream genes (e.g., PAL-1, PAL-2, BGLU44) that governed the differential accumulation of downstream metabolites. Key environmental drivers included elevation (Elev) and the mean temperature of the warmest quarter (Bio10), which modulated coumarin variation, while solar radiation in May (Srad5) and precipitation seasonality (Bio15) predominantly affected gene expression. Furthermore, a quantitative model linking growth environments to bioactive molecule profiles was established, with the optimal parameters defined as follows: mean annual temperature (Bio1) = 9.28 °C, annual precipitation (Bio12) = 1483.66 mm, elevation = 1765.08 m, total solar radiation (SradSUM) = 152,833.23 kJ/m^2^/d, and soil organic carbon (S_oc) = 11,873.95 mg/kg. This work provides a scientific foundation for the ecological cultivation and sustainable utilization of *A. biserrata* roots, while highlighting the critical role of ecological strategies in medicinal plant conservation. Our findings propose novel interdisciplinary approaches bridging ecology, agriculture, and phytomedicine for optimized resource management.

## Figures and Tables

**Table 1:** Distribution of metabolites detected by metabolomics.

Class	Count	Percent
Benzene and Substituted Derivatives	314	6.04%
Carboxylic Acids and Derivatives	544	10.46%
Coumarins and Derivatives	108	2.08%
Fatty Acyls	707	13.6%
Flavonoids	291	5.6%
Glycerophospholipids	95	1.83%
Organooxygen Compounds	649	12.48%
Prenol Lipids	462	8.88%
Steroids and Steroid Derivatives	134	2.58%
Others	1896	36.46%

**Table 2:** The proportion of differential metabolite classifications in each comparison group.

	C-VS-H	G-VS-C	S-VS-C	G-VS-H	G-VS-S	S-VS-H
Organooxygen compounds	18.8%	14%	15.3%	16.6%	13.8%	14.9%
Prenol lipids	8.6%	8.8%	11.3%	8.7%	11.1%	12.6%
Coumarins and derivatives	8.9%	9.2%	6.7%	11.3%	10.4%	9.7%
Carboxylic acids and derivatives	10.2%	10.3%	10.1%	8.7%	7.9%	10%
Fatty acyls	8.9%	10.7%	11.6%	11.7%	10.4%	13%
Flavonoids	8.9%	5.9%	7.3%	7.1%	8.3%	6.7%

**Table 3:** Information on key genes involved in coumarin synthesis and their differential expression.

Enzyme	Numbering	Gene ID	Gene Name	Regulation					
				C-VS-H	G-VS-C	G-VS-H	S-VS-C	S-VS-G	S-VS-H
phenylalanine ammonia-lyase (PAL), K10775	Gene2	TRINITY_DN23454_c1_g2_i2_3	*PAL-3*	Down	UP	Down	UP	Down	Down
	Gene1	TRINITY_DN21310_c0_g2_i1_4	*PAL-1*	Down	UP	Down	UP	Down	Down
4-coumarate--CoA ligas (4CL), K01904	Gene3	TRINITY_DN18825_c0_g1_i2_4	*4CL-1*	Down	UP	Down	UP	Down	Down
	Gene4	TRINITY_DN22443_c1_g2_i2_2	*4CL-1*	Down	UP	Down	UP	Down	Down
	Gene6	TRINITY_DN22443_c1_g4_i1_2	*4CL-2*	Down	UP	Down	UP	UP	Down
	Gene5	TRINITY_DN23319_c1_g4_i1_3	*4CL-1*	Down	UP	Down	UP	UP	Down
trans-cinnamate 4-monooxygenase (CYP73A), K00487	Gene8	TRINITY_DN19642_c0_g1_i1_3	*C4H1*	Down	UP	UP	Down	Down	Down
	Gene7	TRINITY_DN18621_c0_g1_i3_4	*C4H1*	Down	UP	Down	UP	UP	Down
beta-glucosidase (bglB) K05350	Gene9	TRINITY_DN18641_c0_g1_i2_3	*BGLU44*	Down	UP	Down	Down	Down	Down
	Gene10	TRINITY_DN19407_c0_g1_i2_4	*BGLU44*	Down	Down	Down	UP	UP	UP
	Gene12	TRINITY_DN21033_c0_g1_i8_4	*BGLU18*	Down	Down	Down	UP	UP	Down
	Gene11	TRINITY_DN21908_c0_g2_i2_4	*BGLU44*	UP	Down	UP	Down	Down	Down
beta-glucosidase (bglX) K05349	Gene13	TRINITY_DN19157_c0_g1_i1_4	*ANIA_01804*	Down	Down	Down	UP	UP	Down
	Gene14	TRINITY_DN20626_c2_g1_i5_1	*ANIA_01804*	UP	Down	UP	Down	Down	UP
	Gene15	TRINITY_DN20683_c0_g1_i2_3	*XYL4*	Down	Down	Down	Down	UP	Down
	Gene16	TRINITY_DN20767_c0_g1_i18_2	*BXL1*	Down	Down	Down	UP	UP	Down
	Gene17	TRINITY_DN22550_c1_g4_i2_3	*ANIA_02828*	Down	UP	Down	UP	Down	Down

**Table 4:** DNN model accuracy assessment.

	Training Set Metrics	Test Set Metrics
Mean Squared Error (MSE)	0.0025	0.0043
Root Mean Squared Error (RMSE)	0.0495	0.0654
Mean Absolute Error (MAE)	0.0388	0.05
Coefficient of Determination (R^2^)	0.9772	0.9552

## Data Availability

Data are open access and able at https://www.ncbi.nlm.nih.gov/geo/query/acc.cgi?acc=GSE288417 (accessed on 2 April 2025) code will be made available upon request.

## Supplementary Materials

The supporting information can be downloaded at: https://www.mdpi.com/article/10.3390/ijms26083894/s1.
