# Supplementary material for: Exploring Molecular and Genetic Differences in Angelica biserrata Roots Under Environmental Changes
Source: Int J Mol Sci. 2025 Apr 20;26(8):3894. doi: 10.3390/ijms26083894 (PMC12027792; doi:10.3390/ijms26083894)
Supplement: Supplementary file 1 [file ijms-26-03894-s001.zip › ijms-3517075-supplementary.pdf]

Exploring Molecular and Genetic Differences in Angelica pu-bescens Roots Roots Under Environmental Changes

Chaogui Hu, Qian Li*, Xiaoqin Ding, Kan Jiang, Wei Liang

State Key Laboratory of Aridland Crop Science, College of Agronomy, Gansu Agricultural University, Lanzhou 730070, China
* Correspondence:
Corresponding Author
E-mail address: liqian1984@gsau.edu.cn (Qian Li)

1.1. RNA extraction, cDNA library construction and transcriptome analysis

Total RNA extraction was performed using TRIzol reagent. RNA purity and quantification were assessed using a NanoDrop 2000 spectrophotometer (Thermo Scientific, USA), while RNA integrity was evaluated with an Agilent 2100 Bioanalyzer (Agilent Technologies, Santa Clara, CA, USA). Subsequently, the transcriptome libraries were constructed using the VAHTS Universal V6 RNA-seq Library Prep kit. After quality assessment using the Agilent 2100 Bioanalyzer, sequencing was conducted on the Illumina Novaseq 6000 platform. Three biological replicates of each group were prepared for transcriptome analysis.

The Trimmomatic tool was utilized to process the raw fastq-format data (raw reads), removing reads containing ploy-N sequences and those of low quality to obtain clean reads. After adapter and low-quality sequence trimming, the clean reads were assembled into expression sequence tags (contigs) which were then de novo assembled into transcripts using Trinity software. The longest sequence was selected as the Unigene based on sequence similarity and length for further analysis.

1.2. Materials and methods of metabolomics

Methanol, formic acid, water, and acetonitrile were all purchased from CNW Company, L-2-chlorophenylalanine was purchased from Shanghai Hengchuang Biotechnology Co., Ltd., and LysoPC17:0 was purchased from Avanti Company in the United States. All chemicals and solvents are analytical grade or chromatographic grade.

Weigh 60 mg of the sample into a 1.5 mL centrifuge tube, add two small steel balls and 600 μ L of methanol water (V: V=7:3, containing mixed internal standard, 4 μg/mL), pre cool in a refrigerator at -40 ° C for 2 minutes, grind in a grinder (60 Hz, 2 min), sonicate in an ice water bath for 30 minutes, let it stand overnight at -40 ° C, centrifuge at low temperature for 10 minutes (12000 rpm, 4 ° C), draw 150 μ L of the supernatant with a syringe, filter with a 0.22μm organic phase pinhole filter, transfer to an LC injection vial, and store at -8℃. The chromatographic conditions are as follows: chromatography column: ACQUITY UPLC HSS T3 (100 mm x 2.1 mm, 1.8 um); Column temperature: 45 ℃; Mobile phase: A-water (containing 0.1% formic acid), B-acetonitrile; Flow rate: 0.35 mL/min; Injection volume: 3μL. Three biological replicates of each group were prepared for metabolomics analysis.

Prior to pattern recognition, raw data underwent preprocessing using Progenesis QI v3.0

software (Nonlinear Dynamics, Newcastle, UK) for baseline filtering, peak identification, integration, retention time correction, peak alignment, and normalization. Identification analysis was conducted utilizing The Human Metabolome Database (HMDB), Lipidmaps (v2.3), and METLIN databases. To prevent overfitting, the model's quality was evaluated through seven-fold cross-validation and 200 response permutation testing (RPT). RPT is a random permutation method used to assess the accuracy of the OPLS model, ensuring that the classification obtained from the supervised learning method is not random. The OPLS-DA model underwent 200 response permutation tests by fixing the X matrix and permuting the variables in the previously defined classification Y matrix (e.g., 0 or 1) n times (n=200) to establish the corresponding OPLS-DA model and obtain the R2 and Q2 values of the random model. Linear regression was performed on R2Y and Q2Y of the original model, where the intercepts with the y-axis represent R2 and Q2 values, measuring model overfitting. Typically, closer-to-horizontal slopes indicate a higher risk of overfitting; when using RPT testing, Q2 lower than zero is expected in such cases.

1.3. Quantitative real-time polymerase chain reaction (qRT-PCR)

Real-time PCR was performed using LightCycler® 480IIReal-time PCR Instrument (Roche, Swiss) with 10μl PCR reaction mixture that included 1μl of cDNA, 5μl of 2×PerfectStartTM Green qPCR SuperMix, 0.2μl of forward primer, 0.2μl of reverse primer and 3.6μl of nuclease-free water. Reactions were incubated in a 384-well optical plate (Roche, Swiss) at 94℃ for 30s, followed by 45 cycles of 94℃ for 5s, 60℃ for 30s. Each sample was run in triplicate for analysis. At the end of the PCR cycles, melting curve analysis was performed to validate the specific generation of the expected PCR product.

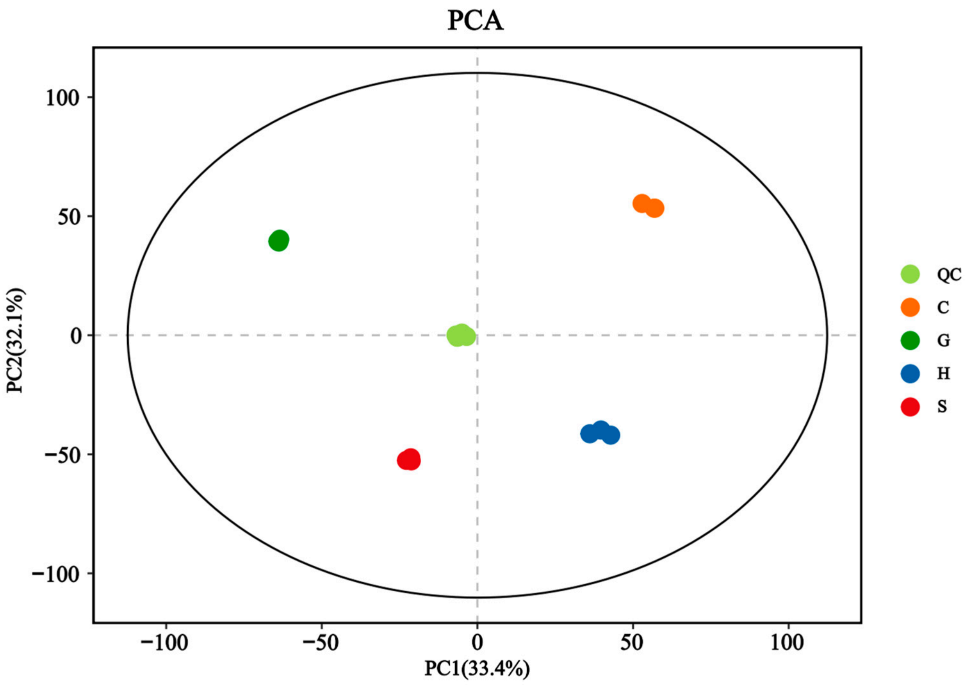

Figure S1. Quality control PCA analysis

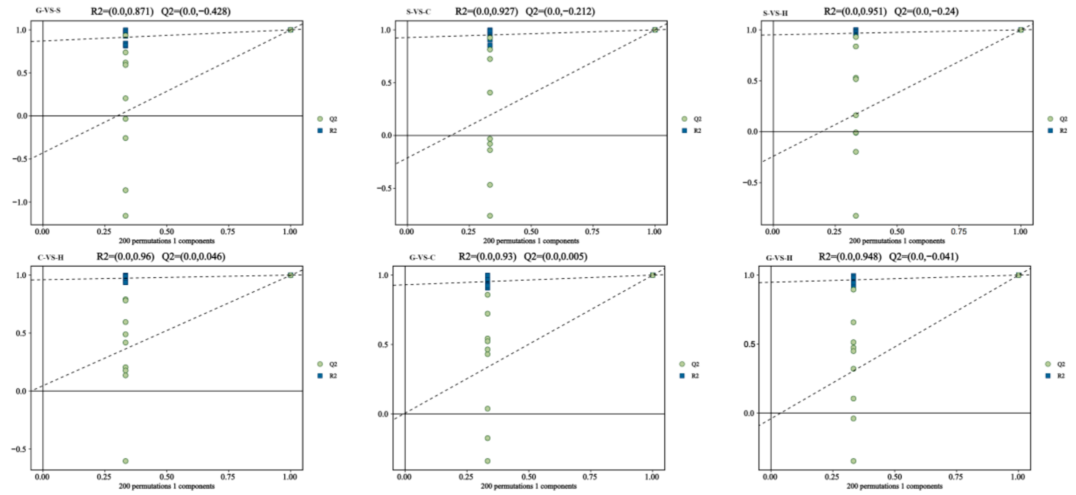

Figure S2. The effectiveness evaluation of the model mainly refers to two criteria: 1. All the green Q2 values on the left are lower than the original points on the right; or 2. The green regression line at point Q2 intersects with the vertical axis (left) at or below zero

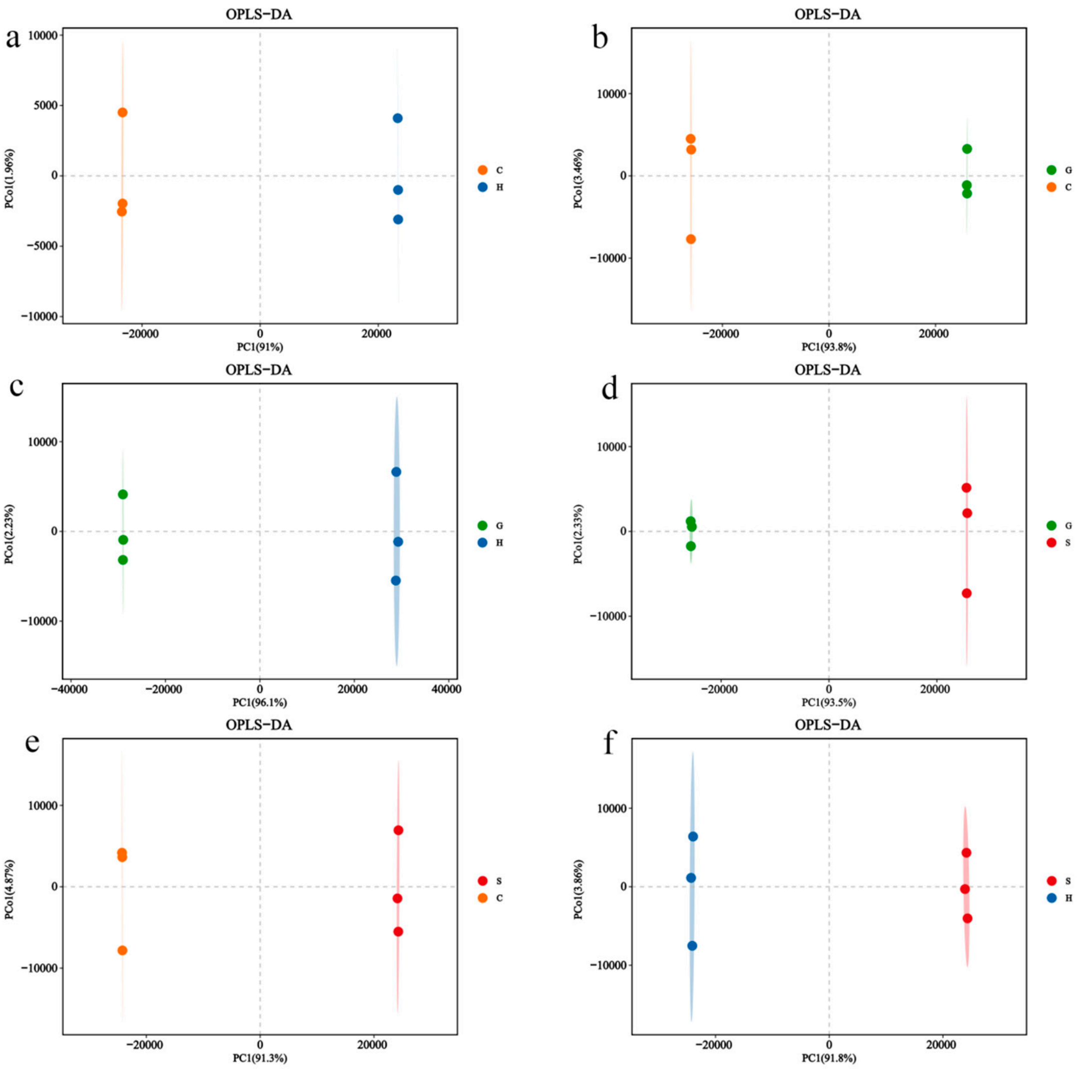

Figure S3. OPLS-DA score map

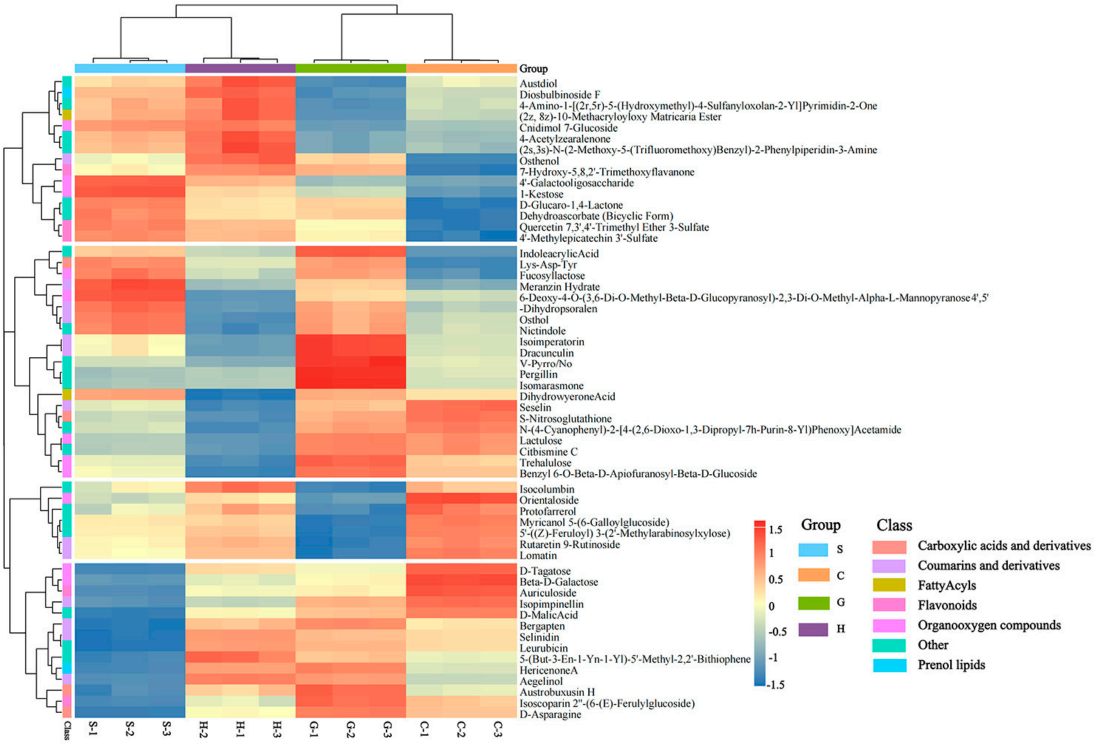

Figure S4. Cluster analysis of Dams

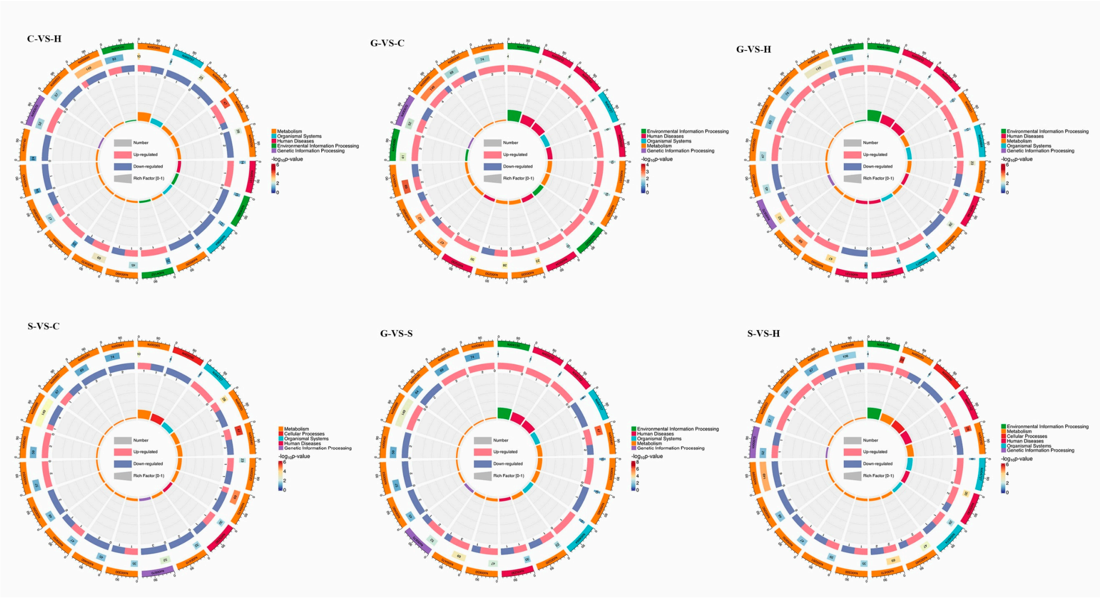

Figure S5. The first circle: Enriched classification, with a coordinate ruler outside the circle representing the number of metabolites, and different colors representing different classifications; Second circle: The number and p-value of this classification in background metabolism. The more metabolites there are, the longer the bar, the smaller the value, the redder the color, and the larger the blue the color; The third circle: A bar chart showing the proportion of upregulated and downregulated metabolites, with light red representing the proportion of upregulated metabolites and light blue representing the proportion of downregulated metabolites; The specific numerical values are displayed below; Fourth circle: RichFactor values for each category, with background auxiliary lines representing 0.2 for each small grid

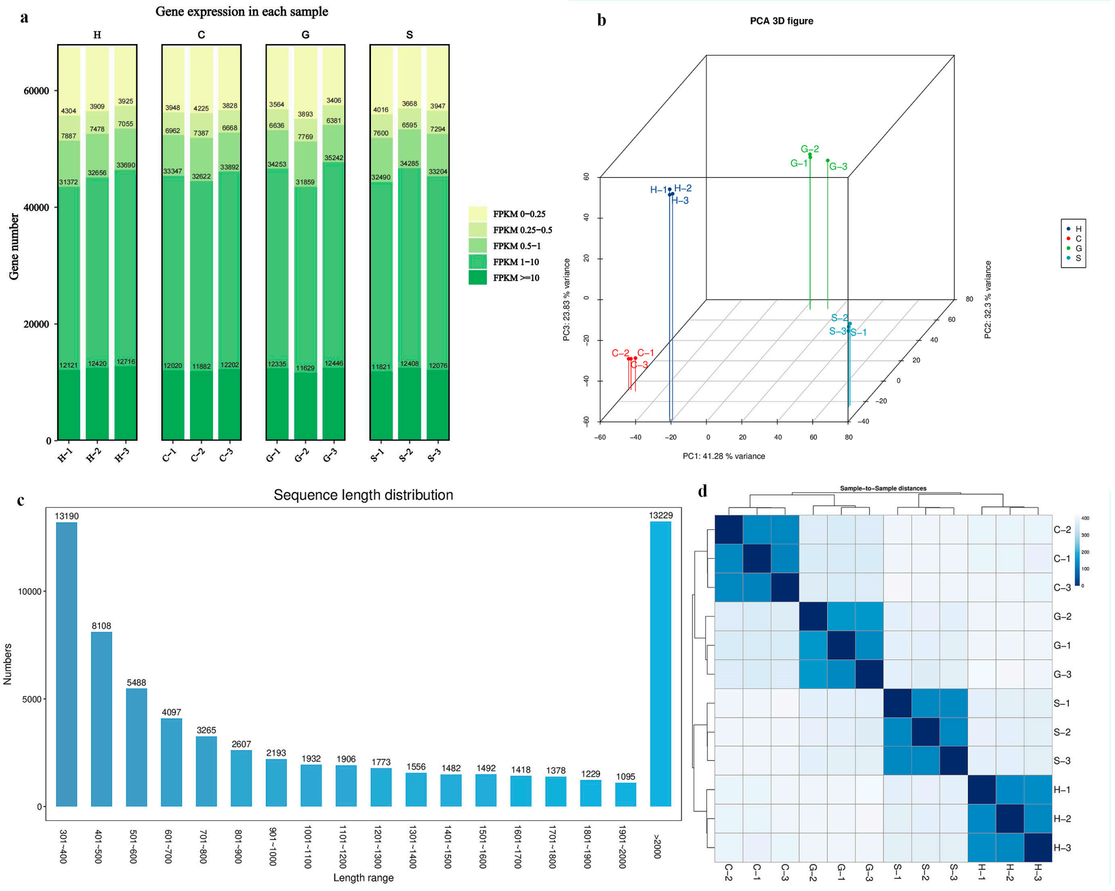

Figure S6 a: FPKM expression distribution range; b: PCA analysis; c: Unigene length distribution; d: sample-to-sample cluster analysis

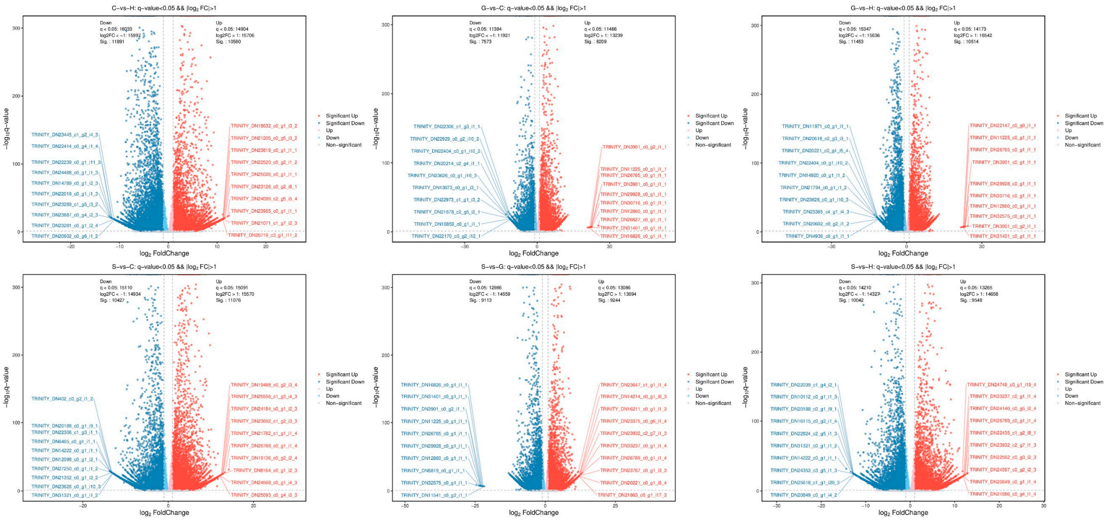

Figure S7. Volcano diagram analysis of DEGs

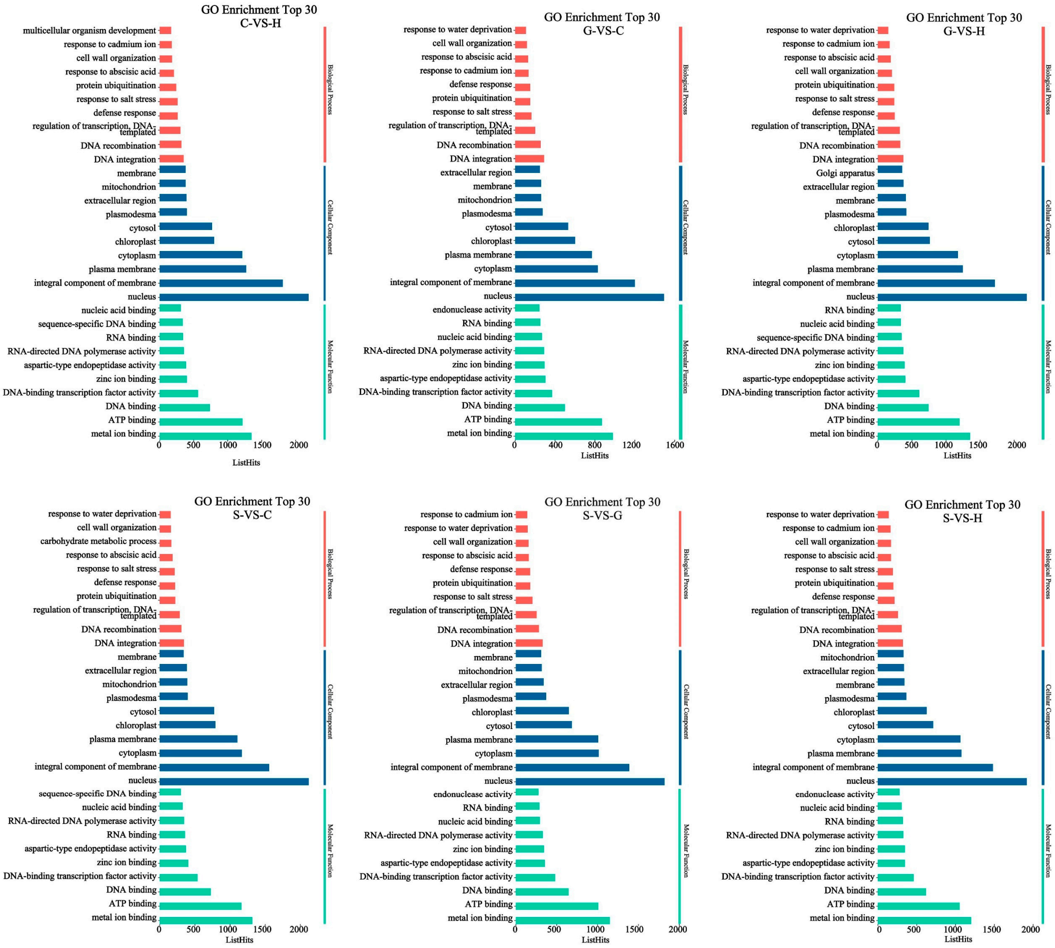

Figure S8. Go enrichment analysis of DEGS

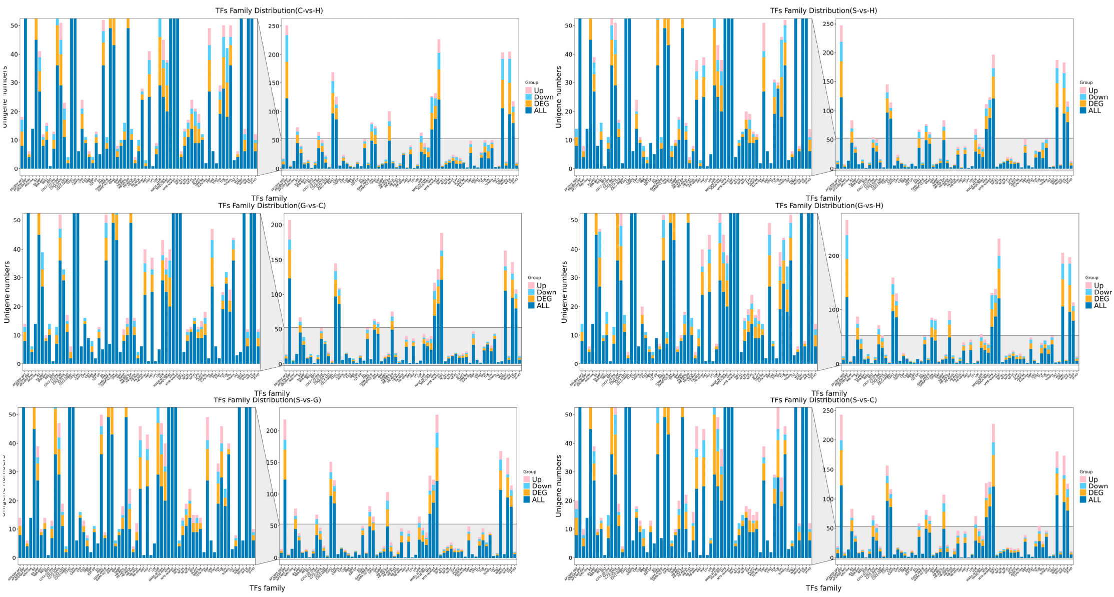

Figure S9. Differential analysis of transcription factors (TFs) distribution

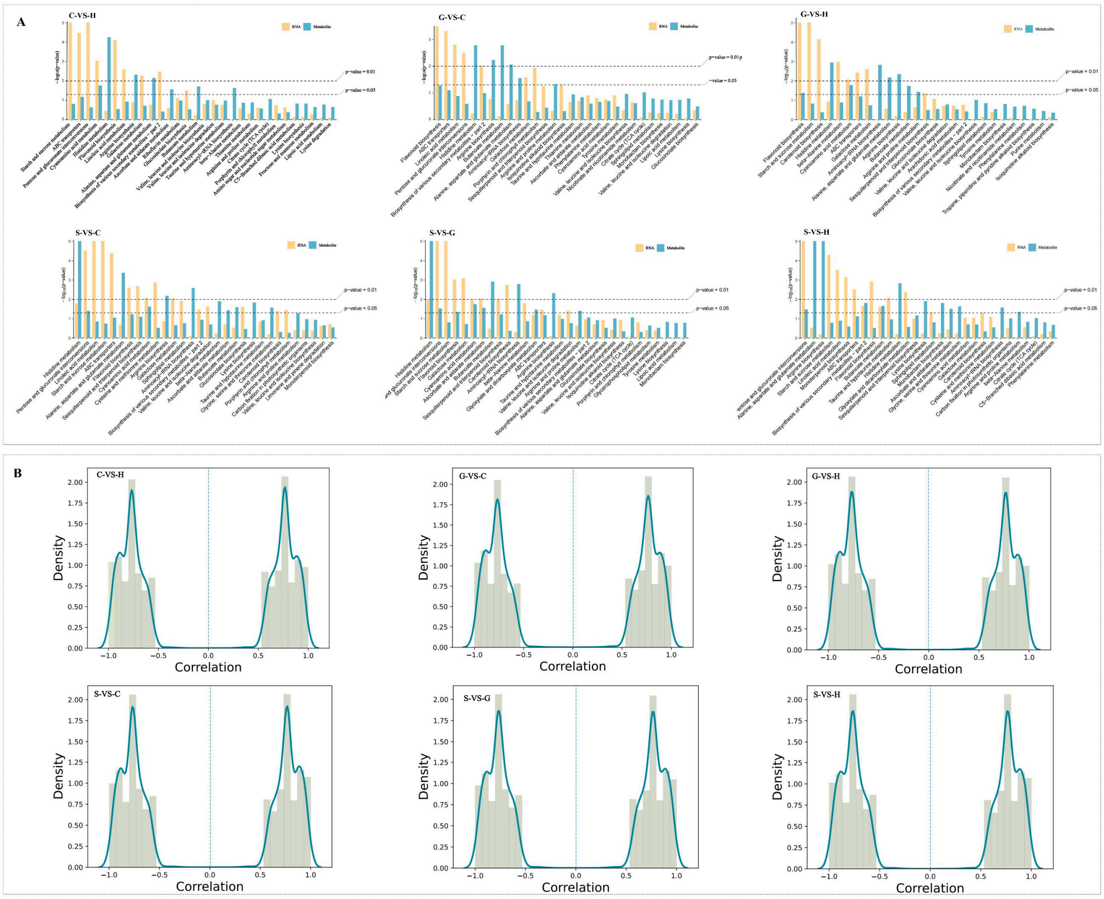

Figure S10. A: Co-enrichment of DAMs and DEGs. B: Correlation concentration between DAMs and DEGs

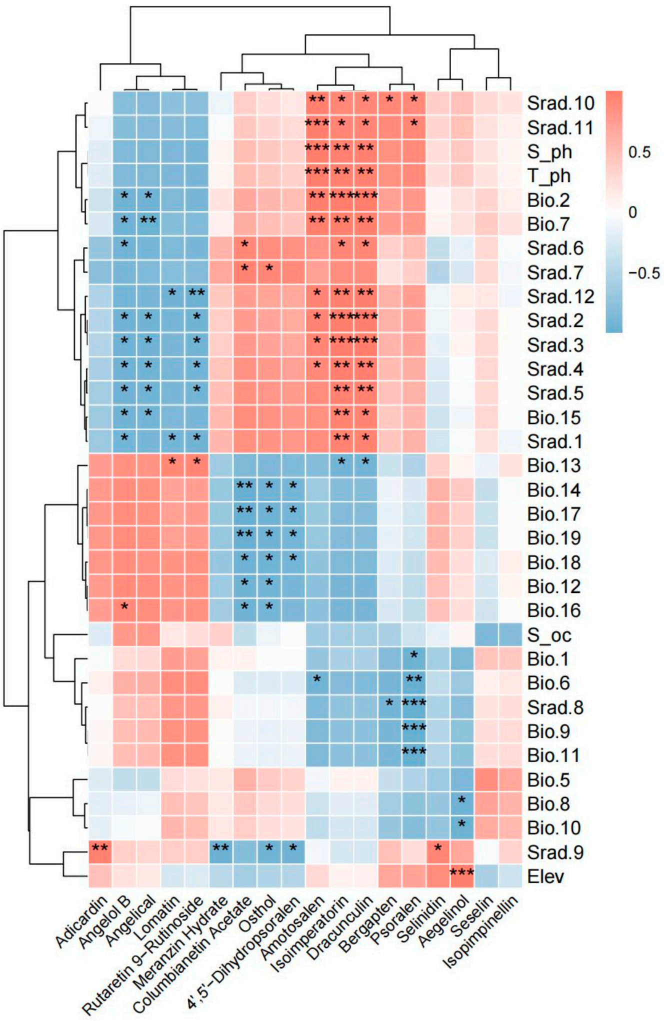

Figure S11. Correlation between environmental variables and coumarin compounds. *: $p < 0.05$; ** : $p < 0.01$; *** : $p < 0.001$.

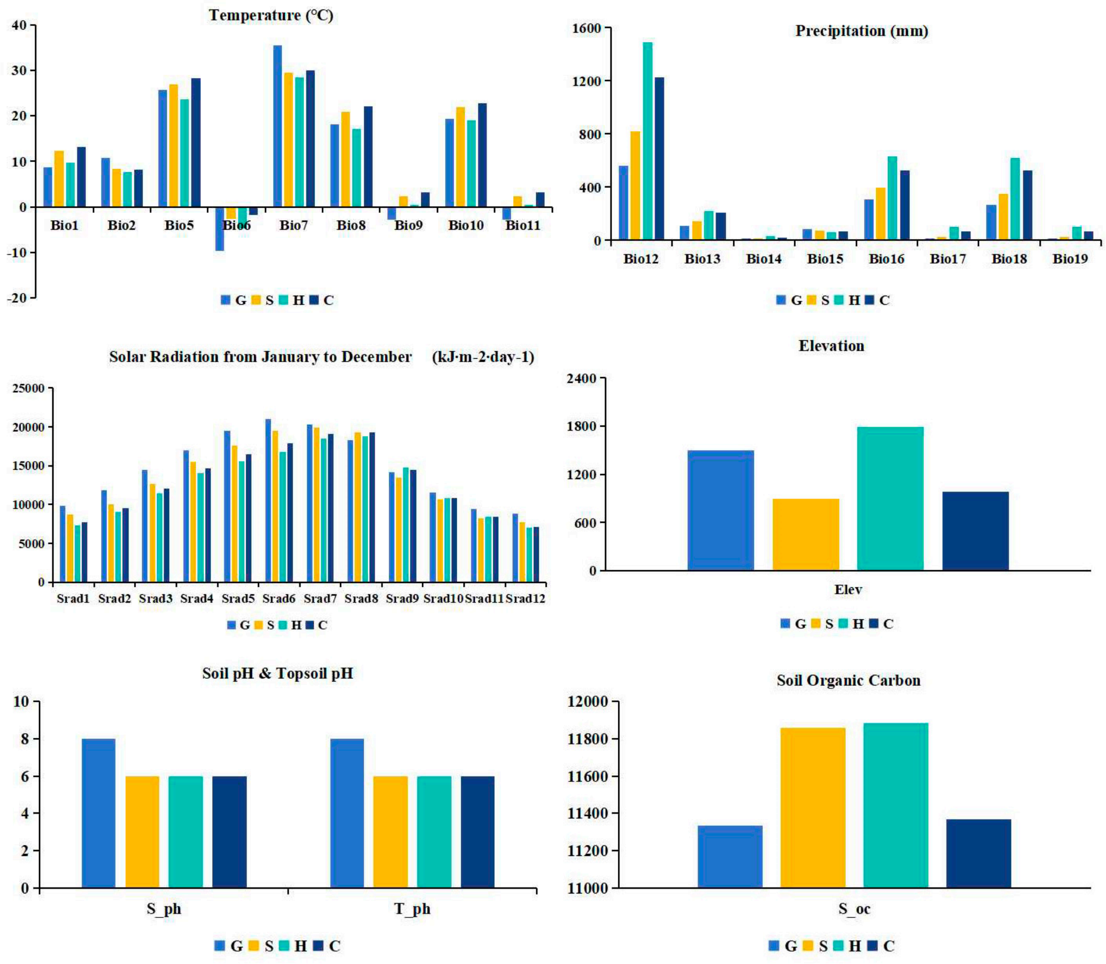

Figure S12. Environmental variable information

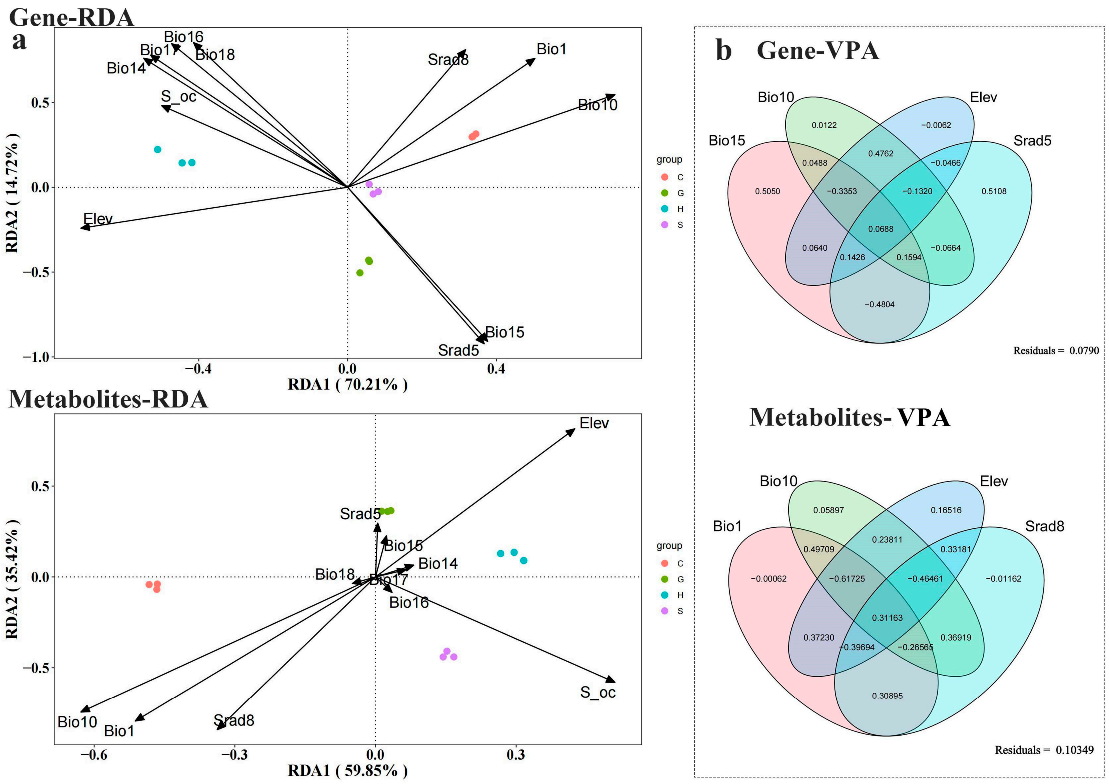

Figure S13. Evaluate the interpretation of environmental variables on metabolites and genes. a: RDA. The above image is a gene, and the following image is a metabolite. b: VPA. The above image is a gene, and the following image is a metabolite.

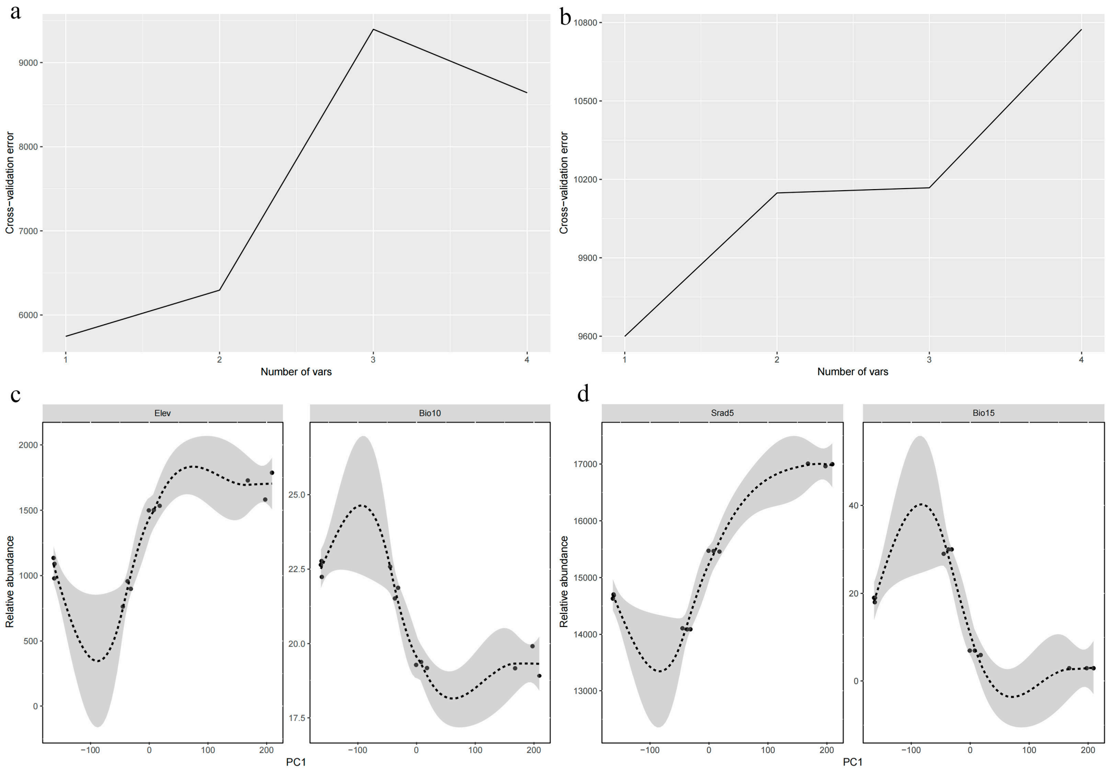

Figure S14. RF analysis of differential metabolites and genes of coumarin by environmental variables. (**a**) Metabolites cross-validation plot; (**b**) Gengs cross-validation plo; (**c**) Metabolites partial dependence plot; (**d**) Gengs partial dependence plot;

Table S1. Primer information of gene

Num	Gene Symbol	Forward primer(5->3)	Reverse primer(5->3)	Product length(bp)	Tm(℃)
1	actin	TGGTATTGTGCTGGATTCTGGT	TGAGATCACCACCAGCAAGG	109	60
2	TRINITY_DN20626_c2_g1_i5_1	TATCGATGCTATTCATGGACAC	TTCTTGACAAGTTCAGGATCTC	99	60
3	TRINITY_DN21970_c0_g1_i3_3	CGTTGCAGATGTTCTCTTCG	ATCGGAAGCTGATCAACATT	84	60
4	TRINITY_DN21310_c0_g2_i1_4	AGAAAGTGACATCGCCTG	AGCTCTGTAGACACTCCAT	96	60
5	TRINITY_DN18621_c0_g1_i3_4	GCTTTGAGTACAATTACGGAGA	CTTGATTTCCTGGCAGAGTT	83	60
6	TRINITY_DN23319_c1_g4_i1_3	ATTGTGGTGCATTGATGGAT	AACCACCCTTCTTCGTCTA	85	60
7	TRINITY_DN19642_c0_g1_i1_3	GGTTTGAGAGCGTAGATGAT	TTGTACTCAAAGCTCTGAGCA	88	60
8	TRINITY_DN18825_c0_g1_i2_4	TCATCCAAACATATCGGATGCT	ACTTCATCTTCAGTGGTCG	114	60
9	TRINITY_DN21908_c0_g2_i2_4	CCAAGTTGAAGGAATGGCT	AGGTTCAAGTCCTGGAGT	82	60

Table S2. Ecological factors variable information

Environment variables	Abbreviation	Unit
Annual Mean Temperature	Bio1	℃
Mean Diurnal Range	Bio2	℃
Isothermality	Bio3	1
Temperature Seasonality	Bio4	1
Max Temperature of Warmest Month	Bio5	℃
Min Temperature of Coldest Month	Bio6	℃
Temperature Annual Range	Bio7	℃
Mean Temperature of Wettest Quarter	Bio8	℃
Mean Temperature of Driest Quarter	Bio9	℃
Mean Temperature of Warmest Quarter	Bio10	℃
Mean Temperature of Coldest Quarter	Bio11	℃
Annual Precipitation	Bio12	mm
Precipitation of Wettest Month	Bio13	mm

Precipitation of Driest Month	Bio14	mm
Precipitation Seasonality	Bio15	mm
Precipitation of Wettest Quarter	Bio16	mm
Precipitation of Driest Quarter	Bio17	mm
Precipitation of Warmest Quarter	Bio18	mm
Precipitation of Coldest Quarter	Bio19	mm
Solar Radiation	Srad(1–12)	$kJ·m^{-2}·day^{-1}$
Topsoil pH	T_pH	1
Topsoil Cation Exchange Capacity	T_cec	$cmol·kg^{-1}$
Soil Cation Exchange Capacity	S_cec	$cmol·kg^{-1}$
Topsoil organic carbon	T_oc	%
Soil Organic Carbon	S_oc	%
AWC Range	Awc_class	1
Altitude	Elev	m

Table S3. KEGG enrichment of DAMs in different comparison groups

Comparison group	Enrichment pathway (id)
C-VS-H	ko00020, ko00040, ko00051, ko00052, ko00053, ko00220, ko00230, ko00250, ko00280, ko00290, ko00300, ko00310, ko00330, ko00340, ko00365, ko00404, ko00410, ko00430, ko00460, ko00470, ko00500, ko00520, ko00540, ko00591, ko00622, ko00630, ko00650, ko00660, ko00720, ko00730, ko00740, ko00785, ko00860, ko00941, ko00960, ko00966, ko00970, ko00980, ko00996, ko00997, ko00998, ko00999, ko02010, ko02060, ko04066, ko04152, ko04727, ko04922, ko04964, ko05131, ko05230
G-VS-C	ko00020, ko00040, ko00052, ko00053, ko00220, ko00230, ko00250, ko00261, ko00280, ko00290, ko00300, ko00310, ko00330, ko00331, ko00340, ko00350, ko00360, ko00365, ko00430, ko00460, ko00470, ko00540, ko00564, ko00590, ko00591, ko00600, ko00623, ko00626, ko00630, ko00633, ko00650, ko00660, ko00720, ko00730, ko00760, ko00785, ko00860, ko00909, ko00941, ko00950, ko00960, ko00966, ko00970, ko00980, ko00996, ko00997, ko00998, ko00999, ko02010, ko04022, ko04024, ko04066, ko04071, ko04080, ko04150, ko04270, ko04664, ko04727, ko04750, ko04922, ko04923, ko04924, ko04964, ko04971, ko04974, ko05012, ko05014, ko05022, ko05032, ko05034, ko05131, ko05142, ko05146, ko05230, ko05231, ko05310
G-VS-H	ko00052, ko00220, ko00230, ko00250, ko00261, ko00280, ko00290, ko00330, ko00331, ko00340, ko00350, ko00360, ko00404, ko00410, ko00460, ko00470, ko00500, ko00540, ko00590, ko00600, ko00623, ko00626, ko00633, ko00650, ko00760, ko00906, ko00909, ko00941, ko00950, ko00960, ko00966, ko00970, ko00998, ko00999, ko02010, ko04080, ko04150, ko04152, ko04664, ko04750, ko04971, ko04974, ko05014, ko05022, ko05131, ko05142, ko05146, ko05230, ko05310
S-VS-C	ko00020, ko00040, ko00052, ko00053, ko00220, ko00230, ko00250, ko00260, ko00261, ko00270, ko00280, ko00290, ko00300, ko00310, ko00330, ko00340, ko00365, ko00404, ko00410, ko00430,

	ko00460, ko00470, ko00500, ko00590, ko00591, ko00600, ko00626, ko00630, ko00650, ko00660, ko00710, ko00720, ko00730, ko00760, ko00770, ko00785, ko00860, ko00902, ko00903, ko00906, ko00909, ko00941, ko00950, ko00960, ko00966, ko00970, ko00980, ko00996, ko00997, ko00998, ko00999, ko02010, ko02030, ko04066, ko04080, ko04152, ko04727, ko04922, ko04964, ko04973, ko04974, ko05131, ko05230
G-VS-S	ko00020, ko00040, ko00052, ko00053, ko00220, ko00230, ko00250, ko00261, ko00280, ko00290, ko00300, ko00310, ko00330, ko00331, ko00340, ko00350, ko00365, ko00404, ko00410, ko00430, ko00460, ko00470, ko00500, ko00564, ko00630, ko00650, ko00660, ko00720, ko00760, ko00785, ko00860, ko00906, ko00909, ko00941, ko00950, ko00960, ko00966, ko00970, ko00997, ko00998, ko00999, ko02010, ko04066, ko04080, ko04150, ko04152, ko04664, ko04727, ko04750, ko04922, ko04964, ko04971, ko04973, ko04974, ko05014, ko05022, ko05131, ko05142, ko05146, ko05230, ko05231, ko05310, ,
S-VS-H	ko00020, ko00040, ko00052, ko00053, ko00220, ko00250, ko00260, ko00261, ko00270, ko00300, ko00310, ko00330, ko00331, ko00340, ko00360, ko00365, ko00410, ko00430, ko00460, ko00470, ko00500, ko00540, ko00590, ko00591, ko00600, ko00622, ko00623, ko00626, ko00630, ko00633, ko00650, ko00660, ko00710, ko00720, ko00760, ko00770, ko00785, ko00860, ko00902, ko00903, ko00906, ko00909, ko00941, ko00970, ko00997, ko00998, ko00999, ko02010, ko02030, ko04066, ko04080, ko04150, ko04727, ko04922, ko04964, ko04973, ko04974, ko05014, ko05022, ko05142, ko05146, ko05230,

Table S4. KEGG enrichment pathway information of DAMS

id	Classification	Enrichment pathway description
ko00020	Metabolism	Citrate cycle (TCA cycle)
ko00040	Metabolism	Pentose and glucuronate interconversions
ko00052	Metabolism	Galactose metabolism
ko00053	Metabolism	Ascorbate and aldarate metabolism
ko00220	Metabolism	Arginine biosynthesis
ko00230	Metabolism	Purine metabolism
ko00250	Metabolism	Alanine, aspartate and glutamate metabolism
ko00261	Metabolism	Monobactam biosynthesis
ko00280	Metabolism	Valine, leucine and isoleucine degradation
ko00290	Metabolism	Valine, leucine and isoleucine biosynthesis
ko00300	Metabolism	Lysine biosynthesis
ko00310	Metabolism	Lysine degradation
ko00330	Metabolism	Arginine and proline metabolism
ko00331	Metabolism	Clavulanic acid biosynthesis
ko00340	Metabolism	Histidine metabolism
ko00350	Metabolism	Tyrosine metabolism
ko00360	Metabolism	Phenylalanine metabolism
ko00365	Metabolism	Furfural degradation
ko00430	Metabolism	Taurine and hypotaurine metabolism
ko00460	Metabolism	Cyanoamino acid metabolism
ko00470	Metabolism	D-Amino acid metabolism
ko00540	Metabolism	Lipopolysaccharide biosynthesis

ko00564	Metabolism	Glycerophospholipid metabolism
ko00590	Metabolism	Arachidonic acid metabolism
ko00591	Metabolism	Linoleic acid metabolism
ko00600	Metabolism	Sphingolipid metabolism
ko00623	Metabolism	Toluene degradation
ko00626	Metabolism	Naphthalene degradation
ko00630	Metabolism	Glyoxylate and dicarboxylate metabolism
ko00633	Metabolism	Nitrotoluene degradation
ko00650	Metabolism	Butanoate metabolism
ko00660	Metabolism	C5-Branched dibasic acid metabolism
ko00720	Metabolism	Carbon fixation pathways in prokaryotes
ko00730	Metabolism	Thiamine metabolism
ko00760	Metabolism	Nicotinate and nicotinamide metabolism
ko00785	Metabolism	Lipoic acid metabolism
ko00860	Metabolism	Porphyrin metabolism
ko00909	Metabolism	Sesquiterpenoid and triterpenoid biosynthesis
ko00941	Metabolism	Flavonoid biosynthesis
ko00950	Metabolism	Isoquinoline alkaloid biosynthesis
ko00960	Metabolism	Tropane, piperidine and pyridine alkaloid biosynthesis
ko00966	Metabolism	Glucosinolate biosynthesis
ko00970	Genetic Information Processing	Aminoacyl-tRNA biosynthesis
ko00980	Metabolism	Metabolism of xenobiotics by cytochrome P450
ko00996	Metabolism	Biosynthesis of various alkaloids
ko00997	Metabolism	Biosynthesis of various other secondary metabolites
ko00998	Metabolism	Biosynthesis of various antibiotics
ko00999	Metabolism	Biosynthesis of various plant secondary metabolites
ko02010	Environmental Information Processing	ABC transporters
ko04022	Environmental Information Processing	cGMP-PKG signaling pathway
ko04024	Environmental Information Processing	cAMP signaling pathway
ko04066	Environmental Information Processing	HIF-1 signaling pathway
ko04071	Environmental Information Processing	Sphingolipid signaling pathway
ko04080	Environmental Information Processing	Neuroactive ligand-receptor interaction
ko04150	Environmental Information Processing	mTOR signaling pathway
ko04270	Organismal Systems	Vascular smooth muscle contraction
ko04664	Organismal Systems	Fc epsilon RI signaling pathway
ko04727	Organismal Systems	GABAergic synapse
ko04750	Organismal Systems	Inflammatory mediator regulation of TRP channels
ko04922	Organismal Systems	Glucagon signaling pathway
ko04923	Organismal Systems	Regulation of lipolysis in adipocytes
ko04924	Organismal Systems	Renin secretion
ko04964	Organismal Systems	Proximal tubule bicarbonate reclamation
ko04971	Organismal Systems	Gastric acid secretion
ko04974	Organismal Systems	Protein digestion and absorption
ko05012	Human Diseases	Parkinson disease

ko05014	Human Diseases	Amyotrophic lateral sclerosis (ALS)
ko05022	Human Diseases	Pathways of neurodegeneration - multiple diseases
ko05032	Human Diseases	Morphine addiction
ko05034	Human Diseases	Alcoholism
ko05131	Human Diseases	Shigellosis
ko05142	Human Diseases	Chagas disease (American trypanosomiasis)
ko05146	Human Diseases	Amoebiasis
ko05230	Human Diseases	Central carbon metabolism in cancer
ko05231	Human Diseases	Choline metabolism in cancer
ko05310	Human Diseases	Asthma

Table S5. Transcriptome sequencing data quality preprocessing results

Sample	RawReads(M)	RawBases(G)	CleanReads(M)	CleanBases(G)	ValidBases(%)	Q30(%)	GC(%)
C-1	48.41	7.11	47.29	6.95	97.7	93.81	42.36
C-2	48.44	7.11	47.31	6.94	97.65	94.2	42.55
C-3	48.11	7.06	46.98	6.89	97.64	94.12	42.01
G-1	41.5	6.1	40.61	5.97	97.87	94.29	42.58
G-2	41.64	6.13	40.83	6.01	98.05	93.94	43.35
G-3	48.8	7.17	47.73	7.01	97.81	94.59	42.35
H-1	47.11	6.95	46.24	6.82	98.16	93.93	43
H-2	47.82	7.03	46.75	6.87	97.76	94.35	42.58
H-3	48.7	7.14	47.5	6.96	97.53	94.07	41.88
S-1	48.48	7.13	47.43	6.97	97.85	94.67	42.9
S-2	48.17	7.07	47.06	6.91	97.69	94.3	42.01
S-3	48.3	7.1	47.22	6.94	97.75	94.11	42.47

Table S6. KEGG enrichment pathway information of DEGS

id	Enrichment pathway description	Classification
ko04075	Plant hormone signal transduction	Environmental Information Processing
ko04626	Plant-pathogen interaction	Organismal Systems
ko00940	Phenylpropanoid biosynthesis	Metabolism
ko04141	Protein processing in endoplasmic reticulum	Genetic Information Processing
ko00500	Starch and sucrose metabolism	Metabolism
ko04016	MAPK signaling pathway - plant	Environmental Information Processing
ko03013	RNA transport	Genetic Information Processing
ko00010	Glycolysis / Gluconeogenesis	Metabolism
ko04144	Endocytosis	Cellular Processes
ko03040	Spliceosome	Genetic Information Processing
ko00270	Cysteine and methionine metabolism	Metabolism
ko04120	Ubiquitin mediated proteolysis	Genetic Information Processing
ko00564	Glycerophospholipid metabolism	Metabolism
ko03015	mRNA surveillance pathway	Genetic Information Processing
ko00520	Amino sugar and nucleotide sugar metabolism	Metabolism

ko00040	Pentose and glucuronate interconversions	Metabolism
ko00230	Purine metabolism	Metabolism
ko00620	Pyruvate metabolism	Metabolism
ko00480	Glutathione metabolism	Metabolism
ko03010	Ribosome	Genetic Information Processing
ko00561	Glycerolipid metabolism	Metabolism
ko04146	Peroxisome	Cellular Processes
ko00190	Oxidative phosphorylation	Metabolism
ko00710	Carbon fixation in photosynthetic organisms	Metabolism
ko00071	Fatty acid degradation	Metabolism
ko03018	RNA degradation	Genetic Information Processing
ko00592	alpha-Linolenic acid metabolism	Metabolism
ko00630	Glyoxylate and dicarboxylate metabolism	Metabolism
ko00640	Propanoate metabolism	Metabolism
ko04070	Phosphatidylinositol signaling system	Environmental Information Processing
ko00052	Galactose metabolism	Metabolism
ko00562	Inositol phosphate metabolism	Metabolism
ko03008	Ribosome biogenesis in eukaryotes	Genetic Information Processing
ko00030	Pentose phosphate pathway	Metabolism
ko00061	Fatty acid biosynthesis	Metabolism
ko00250	Alanine, aspartate and glutamate metabolism	Metabolism
ko03030	DNA replication	Genetic Information Processing
ko04145	Phagosome	Cellular Processes
ko00130	Ubiquinone and other terpenoid-quinone biosynthesis	Metabolism
ko00260	Glycine, serine and threonine metabolism	Metabolism
ko01040	Biosynthesis of unsaturated fatty acids	Metabolism
ko00410	beta-Alanine metabolism	Metabolism
ko00195	Photosynthesis	Metabolism
ko02010	ABC transporters	Environmental Information Processing
ko00860	Porphyrin and chlorophyll metabolism	Metabolism
ko03420	Nucleotide excision repair	Genetic Information Processing
ko00240	Pyrimidine metabolism	Metabolism
ko00280	Valine, leucine and isoleucine degradation	Metabolism
ko00330	Arginine and proline metabolism	Metabolism
ko00051	Fructose and mannose metabolism	Metabolism
ko00900	Terpenoid backbone biosynthesis	Metabolism
ko00511	Other glycan degradation	Metabolism
ko03410	Base excision repair	Genetic Information Processing
ko00941	Flavonoid biosynthesis	Metabolism
ko00400	Phenylalanine, tyrosine and tryptophan biosynthesis	Metabolism
ko00020	Citrate cycle (TCA cycle)	Metabolism
ko00220	Arginine biosynthesis	Metabolism
ko00380	Tryptophan metabolism	Metabolism
ko03440	Homologous recombination	Genetic Information Processing

ko00350	Tyrosine metabolism	Metabolism
ko00460	Cyanoamino acid metabolism	Metabolism
ko00910	Nitrogen metabolism	Metabolism
ko00053	Ascorbate and aldarate metabolism	Metabolism
ko00073	Cutin, suberine and wax biosynthesis	Metabolism
ko04712	Circadian rhythm - plant	Organismal Systems
ko00970	Aminoacyl-tRNA biosynthesis	Genetic Information Processing
ko00906	Carotenoid biosynthesis	Metabolism
ko04136	Autophagy - other	Cellular Processes
ko00591	Linoleic acid metabolism	Metabolism
ko00920	Sulfur metabolism	Metabolism
ko00565	Ether lipid metabolism	Metabolism
ko00905	Brassinosteroid biosynthesis	Metabolism
ko00945	Stilbenoid, diarylheptanoid and gingerol biosynthesis	Metabolism
ko03060	Protein export	Genetic Information Processing
ko00904	Diterpenoid biosynthesis	Metabolism
ko00290	Valine, leucine and isoleucine biosynthesis	Metabolism
ko00360	Phenylalanine metabolism	Metabolism
ko00590	Arachidonic acid metabolism	Metabolism
ko00600	Sphingolipid metabolism	Metabolism
ko03020	RNA polymerase	Genetic Information Processing
ko00100	Steroid biosynthesis	Metabolism
ko00513	Various types of N-glycan biosynthesis	Metabolism
ko00780	Biotin metabolism	Metabolism
ko00062	Fatty acid elongation	Metabolism
ko00340	Histidine metabolism	Metabolism
ko00510	N-Glycan biosynthesis	Metabolism
ko00531	Glycosaminoglycan degradation	Metabolism
ko00563	Glycosylphosphatidylinositol (GPI)-anchor biosynthesis	Metabolism
ko00650	Butanoate metabolism	Metabolism
ko00908	Zeatin biosynthesis	Metabolism
ko00909	Sesquiterpenoid and triterpenoid biosynthesis	Metabolism
ko03022	Basal transcription factors	Genetic Information Processing
ko00450	Selenocompound metabolism	Metabolism
ko00310	Lysine degradation	Metabolism
ko00740	Riboflavin metabolism	Metabolism
ko00760	Nicotinate and nicotinamide metabolism	Metabolism
ko00950	Isoquinoline alkaloid biosynthesis	Metabolism
ko03050	Proteasome	Genetic Information Processing
ko04933	AGE-RAGE signaling pathway in diabetic complications	Human Diseases
ko03430	Mismatch repair	Genetic Information Processing
ko00730	Thiamine metabolism	Metabolism
ko00902	Monoterpenoid biosynthesis	Metabolism
ko04130	SNARE interactions in vesicular transport	Genetic Information Processing

ko00670	One carbon pool by folate	Metabolism
ko00790	Folate biosynthesis	Metabolism
ko00430	Taurine and hypotaurine metabolism	Metabolism
ko00603	Glycosphingolipid biosynthesis - globo and isoglobo series	Metabolism
ko00604	Glycosphingolipid biosynthesis - ganglio series	Metabolism
ko00770	Pantothenate and CoA biosynthesis	Metabolism
ko00903	Limonene and pinene degradation	Metabolism
ko00966	Glucosinolate biosynthesis	Metabolism
ko00196	Photosynthesis - antenna proteins	Metabolism
ko00261	Monobactam biosynthesis	Metabolism
ko00300	Lysine biosynthesis	Metabolism
ko00750	Vitamin B6 metabolism	Metabolism
ko00960	Tropane, piperidine and pyridine alkaloid biosynthesis	Metabolism
ko00998	Biosynthesis of various secondary metabolites - part 2	Metabolism
ko00440	Phosphonate and phosphinate metabolism	Metabolism
ko00965	Betalain biosynthesis	Metabolism
ko03450	Non-homologous end-joining	Genetic Information Processing
ko00072	Synthesis and degradation of ketone bodies	Metabolism
ko00514	Other types of O-glycan biosynthesis	Metabolism
ko00660	C5-Branched dibasic acid metabolism	Metabolism
ko04122	Sulfur relay system	Genetic Information Processing
ko00785	Lipoic acid metabolism	Metabolism
ko01502	Vancomycin resistance	Human Diseases
ko00232	Caffeine metabolism	Metabolism
